# YAP activation is robust to dilution†

**DOI:** 10.1039/d4mo00100a

**Published:** 2024-08-14

**Authors:** Ian Jones, Mar Arias-Garcia, Patricia Pascual-Vargas, Melina Beykou, Lucas Dent, Tara Pal Chaudhuri, Theodoros Roumeliotis, Jyoti Choudhary, Julia Sero, Chris Bakal

**Affiliations:** a Chester Beatty Laboratories, Division of Cancer Biology, Institute of Cancer Research 237 Fulham Road London SW3 6JB UK dmcbijo@ucl.ac.uk; b Institute for Mathematical Innovation, Department of Life Sciences, University of Bath Claverton Down Bath BA2 7AY UK

## Abstract

The concentration of many transcription factors exhibits high cell-to-cell variability due to differences in synthesis, degradation, and cell size. Whether the functions of these factors are robust to fluctuations in concentration, and how this may be achieved, is poorly understood. Across two independent panels of breast cancer cells, we show that the average whole cell concentration of YAP decreases as a function of cell area. However, the nuclear concentration distribution remains constant across cells grouped by size, across a 4–8 fold size range, implying unperturbed nuclear translocation despite the falling cell wide concentration. Both the whole cell and nuclear concentration was higher in cells with more DNA and CycA/PCNA expression suggesting periodic synthesis of YAP across the cell cycle offsets dilution due to cell growth and/or cell spreading. The cell area – YAP scaling relationship extended to melanoma and RPE cells. Integrative analysis of imaging and phospho-proteomic data showed the average nuclear YAP concentration across cell lines was predicted by differences in RAS/MAPK signalling, focal adhesion maturation, and nuclear transport processes. Validating the idea that RAS/MAPK and cell cycle regulate YAP translocation, chemical inhibition of MEK or CDK4/6 increased the average nuclear YAP concentration. Together, this study provides an example case, where cytoplasmic dilution of a protein, for example through cell growth, does not limit a cognate cellular function. Here, that same proteins translocation into the nucleus.

## Abstract 2

Despite dilution of YAP across the whole cell with increasing size, the nuclear concentration remained constant across sizes. YAP activation is robust to whole cell dilution.

## Graphical abstract

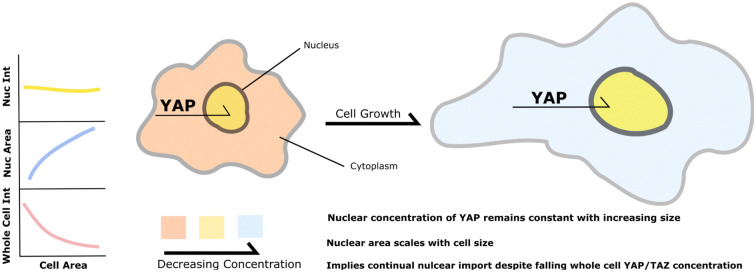

Signalling networks couple transcriptional regulation to the integrated detection of environmental cues. A common ‘motif’ in these networks is the sequestration of transcription factors by inhibitory complexes. In the presence of an environmental cue, transcription factors are released from these complexes and interact with DNA to engage specific programmes.^1–3^ When TFs and inhibitors are at sub-saturating levels, this allows transcription to be tightly coupled to environmental flux. For example, dilution of the inhibitory protein RB1 as cells grow is one mechanism by which E2F activity is coupled to size.^4,5^ In animal cells especially, inhibitory sequestration of transcription factors often occurs in the cytoplasm; and release from inhibition allows TFs to translocate into the nucleus. For example, the active degradation of inhibitors such as APC or IκB in the cytoplasm allows the translocation of transcription factors such as beta-catenin or NFκB into the nucleus;^6,7^ coupling cues such as adhesion and stress to transcription respectively. The concentration of both inhibitors and transcription factors in the cytoplasm thus informs the response of transcription factors to upstream signals.^4^

YAP is a key regulator of animal cell growth and proliferation. As a transcriptional co-activator, YAP is inhibited by sequestration in the cytoplasm, where nuclear translocation (activation) is triggered by soluble, mechanical, and geometrical cues.^8–16^ For example, changes in cell shape are coupled to the signalling dynamics of Rho GTP exchange factors (RhoGEFs), Rho GTPase activating proteins (RhoGAPs) and their downstream effector Rho GTPases. RhoGEFs, RhoGAPs, and Rho GTPases regulate YAP translocation both by regulating YAP signalling directly and by affecting cell shape/size (indirectly).^9,13,17–23^ The coupling of YAP dynamics to cell shape provides a mechanism that allows cells to position themselves during development, or to sense and respond to disruption in tissue structures.^17,24–26^ YAP is also regulated by the Hippo pathway, whereby the LATS (LATS1 and LATS2) kinases phosphorylate YAP, preventing nuclear translocation by enabling 14-3-3 binding,^27^ and promoting sequestration to tight junctions through AMOT phosphorylation.^28^ YAP may be further regulated *via* having no canonical nuclear localisation signal of its own, instead relying on co-transport as part of a complex, or binding partners in the nucleus to encourage nuclear retention, such as MAML.^29^ Note this does not appear to be the case in *Drosophila*, where residues 1–55 of Yki function as a makeshift NLS, being required for nuclear translocation and directly interacting with importin-a1.^30^

It is now clear that the concentration of many cellular molecules varies between cells, even within an isogenic population.^31^ Such variability can be due to both extrinsic and intrinsic stochastic differences in protein synthesis, but also due to differences in cell size and shape.^31–34^ However, cell populations and tissues exhibit largely robust and predictable behaviour despite such fluctuations. Perhaps the best example of which is the control of size uniformity during proliferation, such as during organ and tissue morphogenesis.^35–37^ But how signalling events are robust to fluctuations in protein concentration is poorly understood.

In the context of a sub-scaling protein, one means to provide robustness would be to couple a synthesis step to cell growth, such that a surge in synthesis offsets the effects of dilution. Indeed, such a system is believed to underpin the maintenance of RB1 (yeast WHI5) concentration across consecutive cycles.^4^ In the RB1/WHI5 context, this coupling is complemented by saturating DNA with RB1/WHI5 (and degrading the excess) prior to division;^38^ where the amount of protein inherited by either daughter is proportional to the DNA content – and thus size – of the cell. Indeed, a similar system applies to the partitioning of KRP4 in Arabidopsis.^39^ Importantly, these systems can do nothing to constrain the effects of dilution either side of this synthesis step; the effects of dilution in these cycle positions, and how they may be mitigated are largely unknown.

Through quantitative analysis of 100 000's of single cells, from 17 cell lines, we have demonstrated that the whole cell and cytoplasmic YAP concentration sub-scales with cell area. Crucially, we observed that the nuclear YAP concentration distribution was constant across the population when binned by cell area, implying continual nuclear import in the background of depleting whole cell YAP concentration. Interestingly, YAP synthesis was upregulated near S-phase. Suggesting that cell cycle plays a role in regulating total YAP levels in cells in response to cell growth in G1.

Through integrative analysis of proteomic data, we found that YAP nuclear transport is predicted by the phosphorylation state of RAS/MAPK, focal adhesion, and nuclear transport components; suggesting a role for these systems in coupling cell/cytoplasm size to nuclear import. Indeed, perturbation of MAPK signalling affected nuclear YAP but did not influence whole cell YAP dilution. Furthermore, a similar effect was observed for or CDK4/6 inhibition, suggesting the nuclear YAP distribution is influenced prior to cycle commitment. This may limit the influence of a diluting whole cell concentration in subsequent cycle phases. Taken together, our work begins to explore the robustness of cell signalling to changes in protein concentration, for example, driven by changes to cell shape and size.

## 1. Results

### A. YAP concentration decreases with increasing cell area

**Fig. 1:**
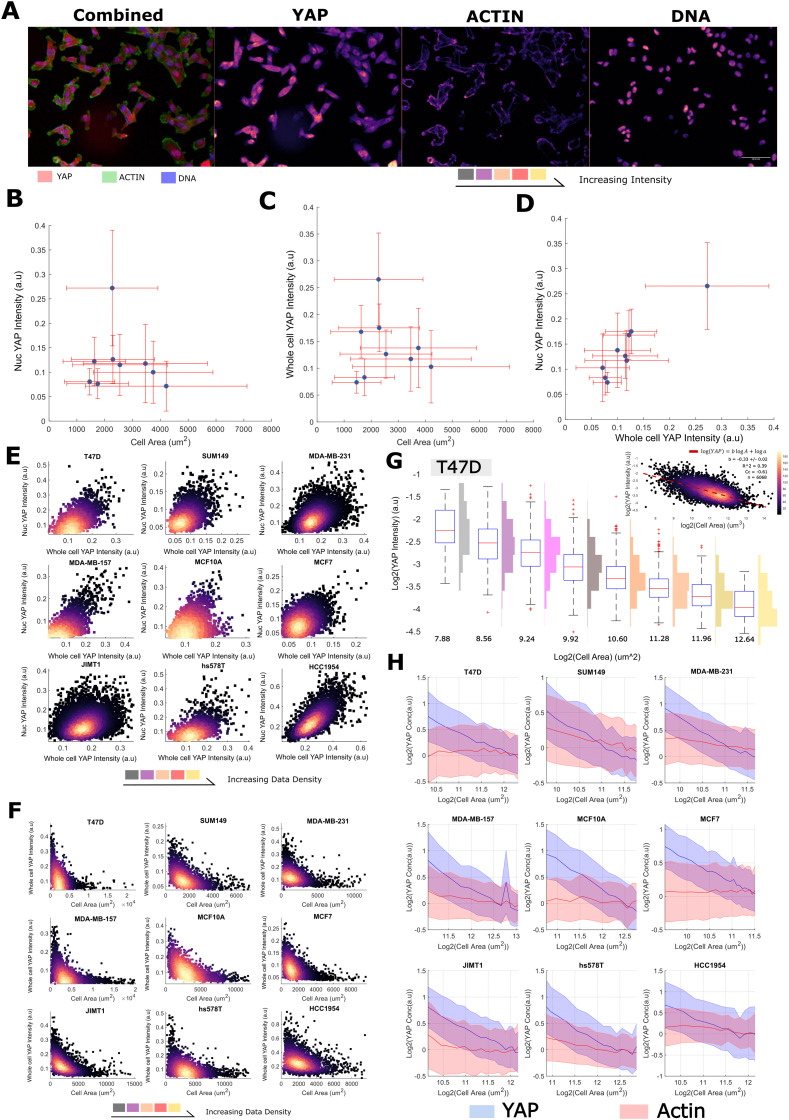
YAP concentration decreases with increasing cell size (A) (left) representative image of MDA-MB-231 cells stained with YAP (red), phalloidin (an actin binding dye, green) and Hoescht (marking the DNA in blue). (right) Individual colour channels seperated out from the original image. Colour is proportional to image intensity such that black < purple < red < yellow. The scale bar represents 50 μm. (B) The relationship between the nuclear YAP concentration and cell area across cell lines. Error bars represent one standard deviation. (C) The relationship between the whole cell YAP concentration and cell area across cell lines. Error bars represent one standard deviation. (D) The relationship between the whole cell YAP concentration and the nuclear YAP concentration across cell lines. Error bars represent one standard deviation. (E) The relationship between the whole cell YAP concentration and the nuclear YAP concentration in single cells in each cell line. The colour is proportional to data density such that black < purple < red < yellow. (F) The relationship between the whole cell YAP concentration and cell area in single cells in each cell line. The colour is proportional to data density such that black < purple < red < yellow. (G) Log–log relationship between YAP ‘concentration’ (intensity) and cell area in T47D cells. Histograms denote the distribution of whole cell YAP intensity across cells in the corresponding log_2_(cell-area) bin. The upper right panel shows the same relationship continuously across areas, with the colour being proportional to data density as before. (H) Log–log plots relating YAP (blue) and Actin (red) concentration to single-cell area. The line represents the mean YAP/Actin concentration in each size range. The error margin corresponds to one standard deviation in the same size bin. Concentrations have been normalised to the means across all sizes for viewability. For each cell line, the relationship is shown across the size range: 0.5 × mean – 2 × the mean cell size.

We quantified single cell size and the concentration/abundance distributions of F-actin and cytoplasmic/nuclear YAP, first in 30 000 single cells from nine breast cell lines (Table 1 and Fig. 1A) leveraging a fixed high content imaging dataset generated in a prior study.^14,40^ We initially investigated whether average YAP intensities varied with cell area across our panel but found no evidence of a correlation with the nuclear or whole cell signal (Fig. 1B and C). However, there was a clear linear relationship between the whole cell and nuclear YAP mean intensities (Fig. 1D). Thus high expression of YAP correlates with more nuclear import. This relationship was also observed in single cells within each cell line (Fig. 1E). Strikingly, when investigating whether area predicts whole cell YAP in single cells, we observed a clear negative correlation. Meaning that whole cell YAP dilutes/degrades as the cells grow/spread (Fig. 1F). This prompted us to more formally investigate the decrease of whole cell YAP within each cell line.

**Table 1:** Cell line information: gene cluster: Lu = luminal, BaA = basal A, BaB = basal B. ER/PR/HER2: +/ from protein and mRNA expression; [] inferred from mRNA expression; M = mutant, WT = wild-type. MDA 231-LM2-4175* cells are the highly metastatic subpopulation 4175 from MDA-MB-231^14,41–43^

Cell line	Genetic subtype	ER	PR	Her2
MCF7	Lu	+	[+]	−
T47D	Lu	+	[+]	−
BT474	Lu	+	−	+
SKBR3	Lu	−	[−]	+
HCC1954	BaA	−	[−]	+
MDA-MB-468	BaA	[−]	[−]	−
hs578T	BaB	−	[−]	−
MDA-MB-157	BaB	−	[−]	−
MDA-MB-231	BaB	−	[−]	−
SUM149	BaB	[−]	[−]	−
SUM159	BaB	[−]	[−]	−
MCF10A	BaB	−	[−]	−
JIMT1	Unclassified	−	[−]	+
MDA-MB-231-LM2-4175*				

We modelled the concentrations of YAP (integrated intensity/cell area, mean intensity) as power law relationships with cell area, [YAP] = *aA*^*b*^, such that we could define a ‘scaling factor’, ‘*b*’, for each species (Fig. 1G). In a log–log plot, ‘*b*’ is given as the gradient and log(*a*) is the *y*-intercept. Negative values of ‘*b*’ correspond to the dilution of the protein with increasing cell area (sub-scaling), 0, linear scaling, and positive values, concentration of the protein with increasing area (super-scaling). Fitting ‘*a*’ and ‘*b*’ values to each cell line's F-actin concentration profile, we observed linear scaling between cell area and F-actin *b* ranging from −0.2 to 0.2, (Fig. 1H and Table S1, ESI†) as observed in previous studies.^4^ However, when applying the same analysis to whole cell YAP concentration, we observed, for all cells lines, a clear sub-scaling relationship between cell area and whole cell YAP concentration (*b* −0.35 to −0.65), indicating that whole cell YAP dilutes as a cell gets larger (Fig. 1G, H and Table S2, ESI†) (power law *R*^2^ typically 0.4–0.6, correlation coefficient in log–log space ≈ (0.6) across cell lines).

To investigate the decrease in whole cell YAP concentration with cell area, we also analysed the abundance (integrated whole cell intensity, rather than mean) -area relationship and observed that the whole cell YAP abundance increases with cell size, but not at a rate sufficient to maintain a constant concentration (*b* ≈ 0.6). Total YAP increased with size at all sizes implying continued net-synthesis (that is, synthesis must be outpacing degradation, Table S3, ESI†). F-actin exhibited 1 : 1 abundance scaling indicating synthesis exactly offsets the effect of cell area on concentration (Table S4, ESI†).

Together, these data reveal that whole cell YAP dilutes with increasing cell area. As YAP increases in abundance with as cells enlarge, this dilution is not due to net degradation of YAP at larger cell sizes, but rather the effect of an expanding cell acting on an insufficient synthesis-degradation balance.

### B. YAP concentration, but not scaling, is sensitive to DNA-content and cell cycle progression

**Fig. 2:**
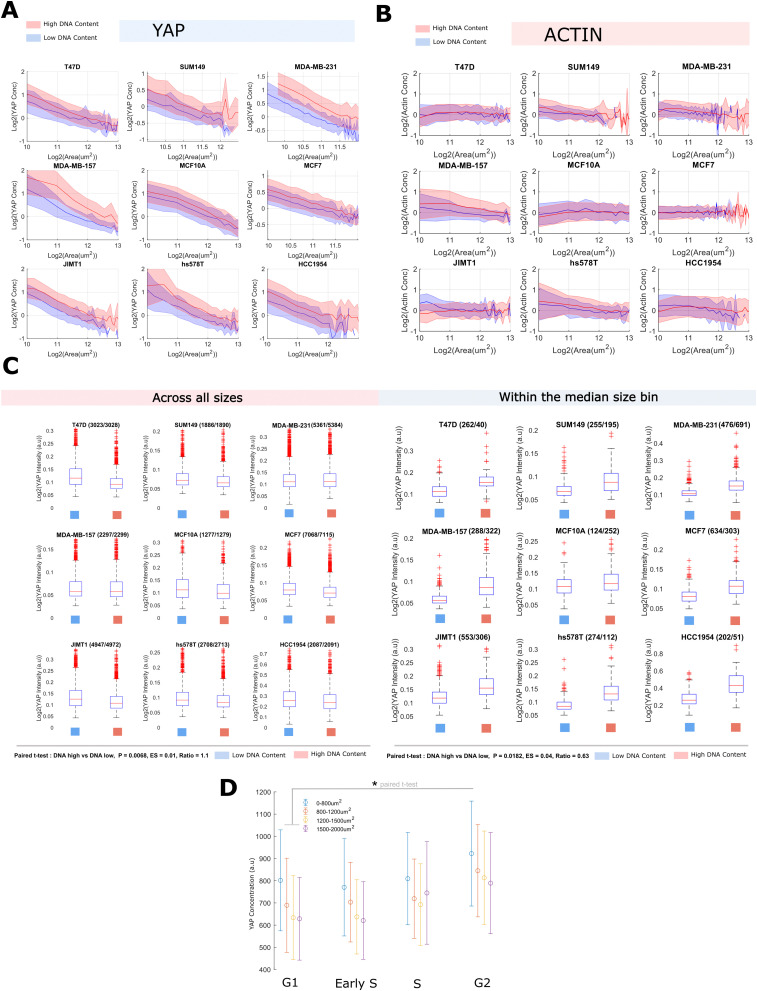
YAP concentration, but not scaling, is sensitive to DNA-content and cell cycle progression (A) log–log plots relating YAP concentration to single-cell area across high and low DNA bins (blue, low bin; DNA < medianDNA, red, high bin DNA > medianDNA). The shaded area denotes one standard deviation of the cell size distribution about that size bin. (B) Log–log plots relating actin concentration to single-cell area across DNA bins (blue, low bin, DNA < medianDNA, red, high bin, DNA > medianDNA). The shaded area denotes one standard deviation of the cell size distribution about that size bin. (C) Relationship between YAP intensity and DNA content (blue, low bin, DNA < medianDNA, red, high bin, DNA > medianDNA) across all sizes (left) or with the median size bin (right). Numbers next to the cell line represent the ‘*n*’ in the DNA low and high groups. (D) YAP intensity across cell cycle phases with four size bins (blue < orange < yellow < purple). ‘*’ Represents *P* < 0.05. Cell cycle stages were dervived from a linear classifer using CycA/PCNA intensity features.

As YAP levels did not correlate with size across lines, but did so within lines, we hypothesised that sub-scaling of whole cell YAP may be due to growth during cell cycle processes. To investigate this, within each line, we binned cells into high and low DNA content groups (integrated Hoechst intensity). Conducting the previous analysis on each DNA ‘bin’ within each line, we noticed that while the mean whole cell YAP concentration at any given size increased (each DNA content ‘bin’, 1.3–1.6 factor increase) (Table S2, ESI† and Fig. 2A, C), the scaling factor ‘*b*’ showed no obvious dependence on the amount of DNA. We performed the same analysis for the F-actin concentration and noticed no relationship between ‘*b*’ or mean concentration and DNA content (Fig. 2B, C and Table S1, ESI†). Whole cell YAP and F-actin abundance showed a consistent positive scaling factor (≈0.4–0.6 YAP, ≈1 actin) across both DNA content groups (Tables S3 and S4, ESI†).

As whole cell YAP dilutes within each DNA ‘bin’, loosely approximating ‘G1’ and ‘G2’, and increases across DNA bins, perhaps emulating S-phase, we sought to more rigorously investigate the relationship between cell cycle progression and YAP concentration. We stained MCF10A cells for YAP, PCNA and CCNA2 and trained a linear classifier to distinguish G0, G1, S and G2 cells using 110 CCNA2 and PCNA intensity features (methods) across 20 000 single cells. This approach follows from PCNA increasing in expression throughout G1–S and G2 while taking on a speckled appearance during S-phase.^44,45^ Thus S-phase may be defined by the spotty-ness of PCNA, and G1 and G2, low/high PCNA expression.^44^ CCNA2 shows a similar upregulation at G2 and acts as a supporting measure in the determination of cell cycle phase,^46,47^ along side measures of DNA content and cell and nuclear size. By binning the cells by area and calculating the mean YAP concentration in each stage, we observed that the whole cell YAP concentration increases from G1 to G2 and that smaller cells exhibit a greater whole cell YAP concentration at each stage, further corroborating the previous analyses (Fig. 2D).

Together, these data show that the regulation of the cytoplasmic YAP concentration is closely tied to the cell cycle, with YAP synthesis being strongly upregulated around S-phase.

### C. A constant nuclear concentration of YAP is maintained across cell areas despite whole cell dilution

**Fig. 3:**
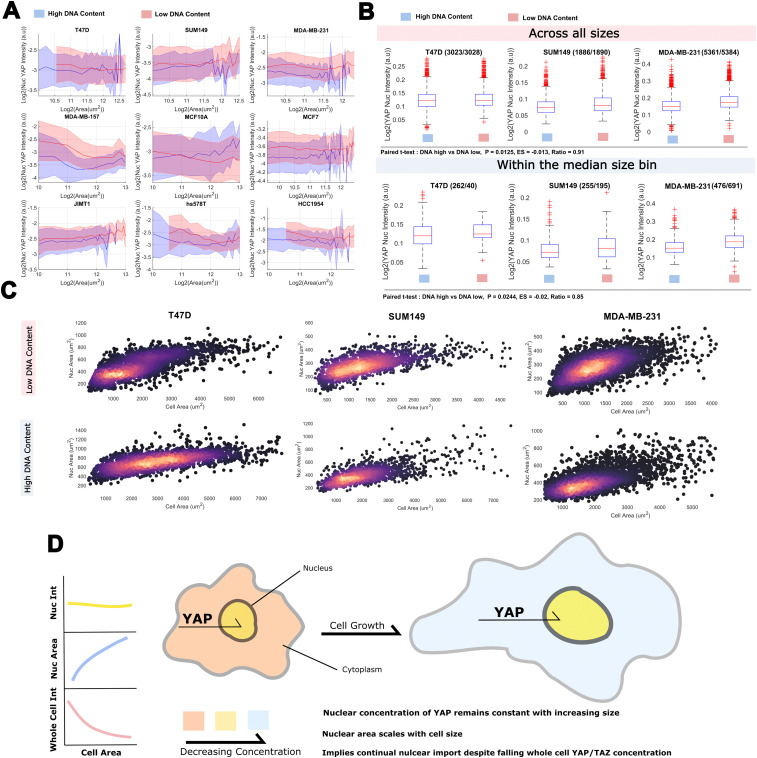
A constant nuclear concentration of YAP is maintained across cell sizes despite whole cell dilution: (A) nuclear YAP, single cell area relationship plotted across DNA content bins for each cell line. The shaded area denotes one standard deviation of the cell size distribution about that size bin (blue, low bin, DNA < medianDNA, red, high bin, DNA > medianDNA). (B) Relationship between nuclear YAP intensity and DNA content (blue, low bin, DNA < medianDNA, red, high bin, DNA > medianDNA) across all sizes (left) or with the median size bin (right). Numbers next to the cell line represent the ‘*n*’ in the DNA low and high groups. (C) Correlation between nuclear and cell area in each DNA bin. Colour is proportional to the density of the data (black < purple < yellow). (D) A cartoon summarising the major findings of the section: nuclear YAP concentration is constant across sizes, nuclear area scales with size, together implying continual YAP nuclear import despite a falling whole cell concentration.

Having observed a sub-scaling relationship between whole cell YAP and cell area, we were interested in how this related to the nuclear translocation and concentration of YAP. Strikingly, the nuclear concentration distribution of YAP was almost entirely insensitive to increases in cell area, exhibiting a constant mean and variance across all measured sizes. Cells with a neighbour fraction (fraction of perimeter touching another cell) greater than 0.7 were excluded from this analysis to prevent cell crowding colouring the result (Fig. S2, ESI†). Increases in the DNA content did increase the average nuclear YAP level, but not sufficiently to maintain the same YAP ratio across DNA bins (Fig. 3A, B and Tables S5, S6, ESI†). Importantly, as nuclear and cell size correlate (Fig. 3C), even without an increase in DNA content, YAP is not being diluted through nuclear growth. Translocation results in a critical YAP concentration regardless of the size of the nucleus. Thus, nuclear transport of YAP is not limited by the decreasing cytoplasmic concentration, or is coupled to cell area to offset the effects of a diluted cytoplasmic/whole cell pool.

Together, these data suggest that while the concentration of cytoplasmic (and whole cell) YAP is a function of cell area, the nuclear YAP concentration is regulated independently. Given the scaling between nuclear and cell area, necessitating a greater import of YAP for the same nuclear concentration, this may be driven by a compensatory upregulation of the nuclear transport machinery (summarised in Fig. 3D).

### D. The nuclear YAP concentration distribution is associated with altered RAS, adhesion and nuclear transport signalling processes

Having observed the dilution of whole cell YAP with increasing size, and that size had no tangible correlation with the nuclear concentration distribution across the population, we reasoned that nuclear transport of YAP was not limited by the cytoplasmic concentration and were interested in how continual and/or equivalent import could be sustained across sizes whilst the cytoplasmic pool depletes. To investigate this, we combined high-throughput imaging and phosphoproteomic experiments across a separate panel of eight cell lines (semi-redundant with the previous panel) (Table 1). The cell lines selected were similar sizes (within a 2-fold range) to prevent size-related phosphorylation events colouring the investigation of nuclear YAP and ratio correlates (Table S7, ESI†). YAP exhibited area sub-scaling behaviour at the whole cell level, but not in the nucleus (relative to tubulin intensity), as is consistent with the prior dataset (Fig. S1, ESI†).

We predicted the cell lines nuclear YAP and N/C ratio from the phosphoproteomic expression data using partial-least squares regression (PLSR). For this, the expressions of each phosphopeptide were ‘corrected’ such that they reflected how much more/less expressed they were than expected given the detected expression of the unphosphorylated peptide (see Methods, Fig. S3, ESI†). This eliminated the trivial correlation between proteomic and phosphoproteomic data and gives information on the signalling state of the cells. From the PLSR model, we could calculate the contribution of each phospho-peptide and thus, how predictive each phosphopeptide was, as achieved through calculation of a ‘variable importance to projection’ (VIP) score.

**Fig. 4:**
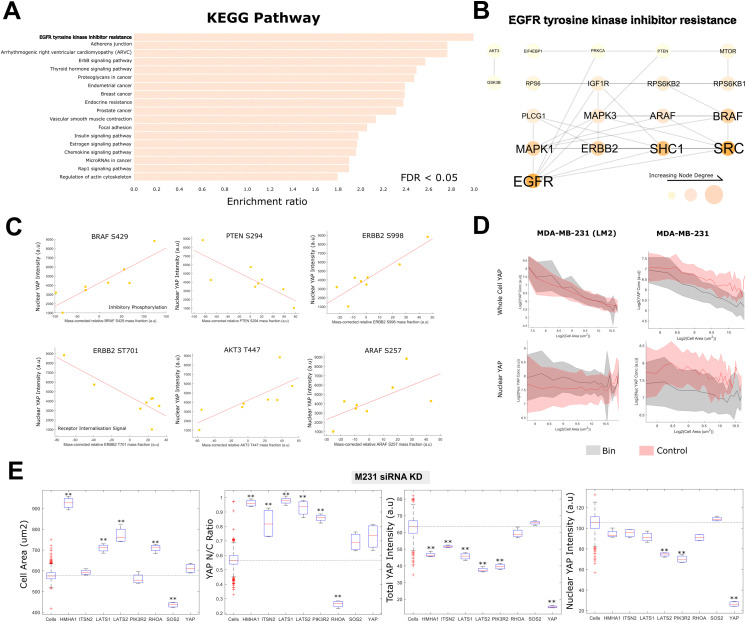
The nuclear YAP concentration distribution is associated with altered RAS, adhesion and nuclear transport signalling processes: (A) themes from the KEGG pathway dataset enriched in the list of phosphopeptides most predictive of a cells nuclear YAP concentration. All enrichments are significant to FDR < 0.05. (B) A network of the interacting members of the phosphopeptides predictive of the nuclear YAP concentration under the ‘EGFR tyrosine kinase inhibitor resistance’ KEGG pathway. Interactions were derived from the STRING database, only experimentally determined physical interactions are shown. Node size, label size and colour are proportional to the node degree. (C) Example relationships between the nuclear YAP concentration and enriched phosphopeptides from the ‘EGFR tyrosine kinase inhibitor resistance’ KEGG pathway. (D) The effect of binimetinib treatment on YAP whole cell and nuclear scaling in LM2 and MDA-MB-231 cells. Binimetinib increased the nuclear concentration of YAP across all sizes in LM2 cells. Binimetinib had the opposite effect in MDA-MB-231 cells although, also decreased cytoplasmic YAP such that the nuc : cytoplasmic ratio still increased. (E) The effects of various siRNA gene KDs on nuclear YAP conc, whole cell YAP conc, cell area and YAP N/C ratio. ‘**’ Denotes *P* < 0.01. *n* = 4 for each individual KD, *n* = 100 for ‘cells’ (control).

Mean nuclear YAP concentrations were predicted by mass corrected expressions of phosphopeptides enriching for RTK/MAPK signalling (KEGG pathway ‘EGFR tyrosine kinase inhibitor resistance’, FDR < 0.05) (Fig. 4A, see Fig. 4B for a protein–protein interaction network of the genes in the ‘EGFR tyrosine kinase inhibitor resistance’ theme, whose phosphorylation were predictive of YAP nuclear intensity). These included several core regulators of the MAPK pathway including: ERBB2 (HER2) (T701), ARAF (S269), SRC (S75), SOS1 (S1178), MAPK1 (T185), MAPK3 (T202), PTEN (S294) and MTOR (S1261) (Fig. 4C). We also observed a clear association between nuclear YAP concentration and focal adhesion signalling (KEGG pathway ‘focal adhesion’, FDR < 0.05), with phosphorylations on TLN (Y70), VCL (S346), PXN (S533), PTK2 (S29), PAK4 (S474), PAK6 (S616), ITGB4 (S1457/4) strongly correlating (|*R*| > 0.7) (Fig. 4A and Fig. S1, ESI†).^59^

Strikingly, when predicting the YAP N/C ratio, we observed differential phosphorylation on multiple regulators of nuclear transport across cell lines with high and low YAP ratios (GO:0051169, ‘nuclear transport’ FDR < 0.05). These included the nucleoporins, NUP133/153/210/35/188/85 and NDC1 (S406), LMNA (S403), the RAN binding proteins RANBP2 (S2280) and 3 (S27), and XPO1 (S1055), a protein recently directly implicated in YAP export from the nucleus (Table 2 and Fig. S1, ESI†).^58^

**Table 2:** Select phosphorylation sites strongly predictive of the average nuclear YAP concentration and/or Nuc/Cyto ratio across lines

Gene	Site	Kinase	Effect	Correlation	Literature
ERBB2	T701	ERK1/2	Receptor internalization inhibition	Negative	48
BRAF	S429	AKT1/3	Inhibits enzyme activity	Positive	49
SRC	S75	CDK5	Multiple processes including degradation and inhibition	Positive	50
MAPK1	T185	EGFR, MEK	Activates enzyme activity	Positive	51
MAPK3	T202	MEK	Activates enzyme activity	Positive	51
SOS1	S1178	ERK2	Grb2 binding	Negative	52
MTOR	S1261	Downstream of PI3K	Induces cell growth	Positive	53
TLN	Y70	EGFR	SH2B1B binding	Positive	54
VCL	S346	RICTOR	Unknown	Positive	55
PAK4	S474	PRKD1	Activates enzyme activity	Positive	56
RANBP2	S2280	CDK1	Localisation signal	Positive	57
XPO1	S11055	NDR1	Activates export function	Positive	58

To investigate the role of EGFR/MAPK signalling in YAP translocation, and validate our phosho-proteomic analysis, we treated two breast lines, MDA-MB-231 and MDA-MB-231-LM2, with binimetinib, a MEK inhibitor. In LM2 cells, binimetinib treatment resulted in an increase in nuclear YAP per cell size whilst having no obvious effect on the scaling of the whole cell YAP concentration implying increased translocation. Conversely, in 231 cells, binimetinib partially reduced nuclear and whole cell YAP levels (Fig. 4D and Fig. S1, ESI†), however, increased the N : C ratio, as in LM2 cells implying increased nuclear import.

We also tested whether a number of cannonical YAP regulators (*e.g.* LATS1/2, RHOA), amongst several RhoGEFs/GAPs implicated in YAP translocation in a previous study^43^ (*e.g.* SOS2, ITSN2), altered the nuclear localisation of YAP in our cells/conditions (leveraging the publicly available dataset generated by our group using a subset of the cell lines used here under the same conditions,^43^ MDA-MB-231 cells were used for this validation). LATS1/LATS2 inhibitory kinases led to an expected increase in the YAP N/C ratio. This was driven by a combination of an increased cell area, driving down cytoplasmic YAP, and increased YAP translocation (Fig. 4E). The nuclear concentration did not increase under these KDs, however, this was due to reduction in total YAP beyond what was expected from the area change alone. RHOA KD drastically decreased the YAP N/C ratio through a reduction in the nuclear YAP concentration. While RHOA depleation increased cell area, this did not lead to a reduction in whole cell YAP. This is likely due to a change in the relationship between cell area and volume under this condition (Fig. 4E).

Together, these sites suggest that enrichment of nuclear YAP is more closely related to ERK/MAPK signalling activation, the maturation of focal adhesions and traditional Hippo/contractility driven mechanisms in these cells, rather than the absolute cytoplasmic concentration and associated cell area/size.

### E. YAP dilution behaviour is conserved

After characterizing the dilution behaviour of YAP in normal and cancerous breast cells, our study was expanded to examine this effect in different cell contexts and assess its generality as a phenomenon. Specifically, we conducted imaging experiments on WM-266-4 and A375 melanoma cells, as well as retinal pigment epithelial cells (RPE-1).

**Fig. 5:**
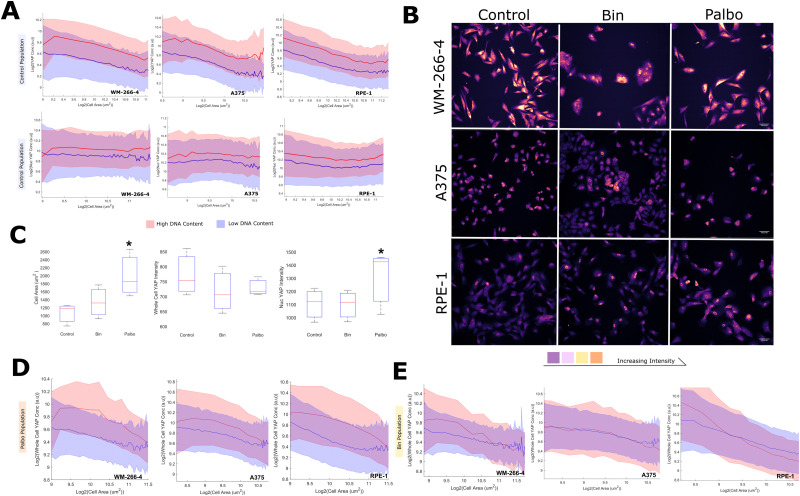
YAP dilution behaviour is conserved across melanoma and RPE cells: (A) (top) The relationship between whole cell YAP intensity and DNA content across control cells. The high DNA content cluster is in red, the low cluster in blue (above and below the median DNA content). The width of the shaded region denotes the standard devition in that size bin. (bottom) The relationship between nuclear YAP intensity and DNA content across control cells. The high DNA content cluster is in red, the low cluster in blue (above and below the DNA content median). The width of the shaded region denotes the standard deviation in that size bin. (B) Representative images of YAP signal across cell lines in each treatment. Colour is proportional to the YAP intensity. (C) Boxplots summarising the effects of palbociclib and binimetinib at the population level on; cell area (left) (*n* = 3 corresponding to each cell line, *P* < 0.05 palbociclib *vs.* control, paired *t*-test), whole cell YAP (ns for any comparison) and nuclear YAP (right) (*P* < 0.05 palbociclib *vs.* control). (D) The relationship between whole cell YAP intensity and DNA content across control cells and palbociclib treated cells. The palbociclib treated population content cluster is in red, the control in blue. The width of the shaded region denotes the standard deviation in that size bin. (E) The relationship between whole cell YAP intensity and DNA content across control cells and binimetinib treated cells. The binimetinib treated population content cluster is in red, the control in blue. The width of the shaded region denotes the standard deviation in that size bin.

Strikingly, across all three of the added lines, YAP dilution was conserved. The YAP concentration, but not the scaling behaviour, was found to be sensitive to DNA content in all cell lines. (Fig. 5A). As in breast cells, the nuclear concentration of YAP remained constant with cell area in the background of whole cell dilution, and this was similarly found sensitive to the DNA content of the cell (Fig. 5A).

We were interested in whether the effects of proliferation/MAPK inhibition similarly extended to the melanoma/RPE contexts and treated each cell line with binemetinib and palbociclib (Fig. 5B). Palbociclib increased the nuclear concentration of YAP in all cases (Fig. 5C). Binimetinib behaved inconsistently across lines, increasing nuclear YAP in WM-266-4 cells, having no effect in A375s and even decreasing nuclear YAP in RPE1 cells. Both treatments consistently increased cell area across the lines, with palbociclib having the more pronounced effect (Fig. 5C). Despite this, neither treatment had any obvious effect on the whole cell YAP concentration (Fig. 5C). This appears to be due to the ‘delay’ of dilution in either treatment, with YAP exhibiting dilution behaviour at larger cell areas under these treatments (Fig. 5D and E). Interestingly, binimetinib treatment appeared to decrease the scaling factor in WM and RPE1 cells (Fig. 5E).

Together, these data show that the YAP dilution phenomenon extends to the melanoma and RPE cell contexts and does not seem to impact the nuclear YAP distribution, evidencing the generality of this effect. CDK 4/6 inhibition increased the nuclear YAP concentration and delayed YAP dilution to larger sizes, implicating cell cycle commitment as a further regulator of nuclear YAP confounding the the influence of the cytoplasmic concentration.

## 2. Discussion

The nuclear YAP concentration distribution did not change across cell size bins suggesting that YAP translocation behaviour is largely constant across small and large cells during proliferation. That is, YAP signalling is robust against changes in whole cell YAP concentration/cell area occurring throughout a division cycle. Such robustness is not a rare phenomenon in biology, indeed, recent works developing models of biological signalling networks have observed remarkably low parameter sensitivity.^60–63^ A particularly striking example can be found in a model of the *Drosophila* segmentation network where, across 48 parameters and two orders of magnitude, if a parameter was assigned a random value, there was a 90% chance that it was associated with a functional network.^64^

Amongst other mechanisms, a biological system may achieve robustness through adaptation.^63^ When investigating the signalling differences in cell lines with high/low average nuclear YAP, we found that the expression of phosphopeptides relating to nuclear transport, adhesion and RTK-MAPK signalling best explained the differences, suggesting that these signalling systems may ‘adapt’ (are up/down regulated with increasing size over generations) to the depleting YAP pool. Indeed, an increased activity of nuclear transporters (and decreased activity of exporters) with increasing size provides an intuitive explanation for how a constant nuclear concentration distribution, sustained by continual import, could be maintained under a falling cytoplasmic concentration. Indeed, XPO1 Ser1055, a phosphopeptide, upregulated in lines with a lower mean nuclear concentration, is an activated species known to control the export of YAP.^58,59^ Furthermore, conformation changes in nuclear pores have been shown to stimulate YAP entry into the nucleus.^65,66^ This can also be driven by increasing nuclear size and thus cell spreading and growth, imparting stress on the nucleus through cytoskeletal connections to the cell body, and may even be sufficient to sustain the nuclear YAP concentration as the cell expands.^67^

Increasing cell volume through growth, where cell constituents are continually synthesised, is distinct from the volume changes associated with cell spreading and osmotic shock, which lead to a cell wide loss of water concentrating/diluting all proteins in the cell equally, and coincides with widespread mechanical signalling events.^68–72^ It is interesting to consider the role of cell size in YAP signalling in these contexts. Indeed, recent work shows osmotic shock with the inert polymer PEG (polyethelene glycol) decreased cell volume, through water loss, and nuclear YAP localisation through a decrease in RAC1 activity,^69^ and another noted decreased YAP activity through AMOT nuclear translocation under hypertonic shock.^73^ Moreover, the related WNT/CTNNB1 signalling system has been shown to be sensitive to volumetric compression, and the resulting molecular crowding, in intestinal organoids.^74^

It would be interesting to study whether the YAP-area/YAP-size relationship holds in more dynamic settings such the onset of cell spreading, where the cell typically increases cell area, but decreases in volume.^68^ When large changes in size and shape occur on short timescales, YAP signalling may be more directly regulated by morphology through the concomitant mechanical alterations to the cell.^13,14^ Indeed, YAP nuclear translocation is famously dependent on cell mechanics, with substrate stiffness and composition,^9,75,76^ focal adhesion signalling^65^ and cell–cell contacts^15^ all being known to regulate YAP signalling events. Recently, many computational tools have been developed to characterise the morphological diversity of cell populations and may provide novel insight into the mechanical and shape dependence of YAP signalling.^77–79^

When perturbing CDK4/6 activity, we observed an increase in the mean nuclear YAP concentration. This may relate to YAP's role in prompting resistance to BRAF-MEK blockade.^80–85^ Binimetinib treatment led to inconsistent responses in the nuclear YAP concentration, but interestingly effected the scaling factor between cell size and whole cell YAP in WM-266-4 and RPE-1 cells. This suggests MAPK signalling may directly contribute to the maintenance of cytoplasmic YAP in these lines, for example by promoting resistance to degradation as observed in *Drosophila*.^86^

It remains unclear whether whole cell YAP dilution effects its cytoplasmic functions; for example, YAP has been shown to influence the spindle assembly checkpoint, potentially through its interactions with BUBR1.^87^ Moreover, cytoplasmic YAP is known to be a core component of the CTNNB1 destruction complex.^88,89^ As YAP and CTNNB1 co-operate as transcription factors in the nucleus,^82,90^ this suggests that the cytoplasmic dilution of YAP may also indirectly influence its nuclear activity in accordance with the putative importance of the YAP nuc/cyto ratio.^91–93^

Together, these data show that that YAP can dilute as the cell increases in size. Remarkably, the nuclear concentration distribution is insensitive to the effect, demonstrating that cell functions are not always linearly related to protein concentration. Thus dilution, for example through cell growth, may not always directly effect cellular functions due to the overlap of multiple potentially limiting systems.

## 3. Materials and methods

### A. Cell culture

The following human breast cell lines were investigated (novel in this study). T-47D and BT-474 were obtained from Nicholas Turner (ICR, London), SKBR3 cells were a kind gift from the laboratory of Olivia Rossanese (ICR), MDA-MB-468 cells were a kind gift from George Poulogiannis (ICR), MDA-MB-231 were obtained from Janine Erler (University of Copenhaguen, Denmark), LM2 cells (a highly metastatic subpopulation 4175 from MDA-MB-231^42^) were obtained from Joan Massagué (Sloan Kettering Institute, New York), while SUM159 were a kind gift from the laboratory of Rachel Natrajan (ICR). All the above cancer cell lines were grown in Roswell Park Memorial Institute (RPMI)-1640 culture medium (Gibco) supplemented with 10% heat-inactivated fetal bovine serum (FBS) and 1% penicillin/streptomycin. MCF10A cells were obtained from ATCC and were engineered to express endogenous mRuby-tagged PCNA.^44^ They were grown in DMEM/F12 supplemented with 5% horse serum, 10 μg ml^−1^ insulin, 20 ng ml^−1^ epidermal growth factor, 100 ng ml^−1^ cholera toxin, 500 ng ml^−1^ hydrocortisone, and 1% penicillin/streptomycin.

All the cell lines were grown at 37 °C and supplemented with 5% CO_2_ in humidified incubators. The passage was carried out using 0.25% trypsin-EDTA (GIBCO) followed by centrifugation (1000 rpm, 4 min) and resuspension in a complete medium. Cell counting was performed using Countess automated cell counter with trypan blue exclusion (Thermo).

Cells were confirmed to be mycoplasma-negative (e-Myco plus Mycoplasma PCR Detection Kit, iNtRON Biotechnology).

WMs, a375, and RPEs cells were maintained in standard culture conditions (DMEM+ 10% FBS, vessel: Corning® Primaria™ 25 cm^2^ Rectangular Canted Neck Cell Culture Flask with Vented Cap, PN: 353808). Passage was carried out using 0.25% trypsin-EDTA (GIBCO) followed by centrifugation (1000 rpm, 4 min) and resuspension in complete medium. Cell counting was performed using Countess automated cell counter with trypan blue exclusion (Thermo).

Prior to imaging/proteomic analysis, cells were plated at day 0 in either 384-well PerkinElmer PhenoPlates (black, optically clear flat-bottom for imaging) or T175cm flasks for proteome analysis. For 384 wells the cell densities used per well were: T-47D (1200 cells), BT-474 (2400 cells), SKBR3 (2200 cells), 468 (1000 cells), 231 (800 cells), LM2 (800 cells), MCF10A (400 cells), and for the proteomics experiments they were scaled according to the surface area of the vessel used. Following three days of incubation in the above growth media, cells were either fixed in pre-warmed 4% formaldehyde (ThermoScientific) in PBS for 15 min at room temperature (image analysis) or collected in a pellet for proteomics analysis.

### B. Immunostaining

After fixation, cells were washed three times in PBS and then permeabilised in 0.2% Triton X-100/PBS solution for 15 min at RT. Following three washes in PBS, cells were blocked for 1 h in 2% bovine serum albumin (BSA) (Sigma)/PBS solution at RT. When using both mouse and rat primary antibodies in the same sample, sequential immunostaining was performed to avoid any antibody cross-reactions. Typically co-immunostaining with a mouse, rat and rabbit antibody was used. After the block step, BSA was removed and the desired mouse primary antibody was added in antibody solution (0.5%BSA/0.01% Triton X-100/PBS) at the indicated dilutions: YAP (G6) (Santa Cruz, 1 : 100), YAP [67.3] (Santa Cruz, 1 : 1000). All the primary antibodies immunostainings were performed overnight at 4 °C. Then cells were washed three times in PBS and incubated with a goat anti-mouse antibody 1 : 1000 in antibody solution for 2 h at RT. Cells were washed three times in PBS and incubated with a rat anti-tubulin alpha antibody (Bio Rad, 1 : 1000) and an anti-rabbit primary antibody when applied, for 2 hours at RT. The anti-rabbit primary antibodies were used at the indicated dilutions: TAZ (V386) (cell signalling, 1 : 200), Anti-PhosphoRB (Abcam, 1 : 1000). Then cells were washed three times in PBS, and incubated for 2 h at room temperature with a goat anti-rat antibody and/or a goat anti-rabbit antibody or Alexa-488 phalloidin (Invitrogen) if needed. Finally, to stain nuclei, 5 mg ml^−1^ Hoescht (Invitrogen)/PBS solution was carried out for 15 min at RT. 384-well plates were sealed for imaging with an Opera Cell:Explorer-automated spinning disk confocal microscope (PerkinElmer) or Opera Phenix (PerkinElmer) in the magnification indicated in the figure legends. At least twenty fields at random positions per well of a 384-well plate were imaged.

For the cell cycle experiments in MCF10A cells, samples were fixed in freshly prepared 4% PFA/PBS for 15 minutes. Cells were subsequently permeabilized with 0.25% Triton/PBS for 10 min and blocked with 0.5% BSA/0.02% glycine/PBS for 30 minutes. Primary antibodies CCNA2 (Abcam, ab181591, 1 : 250) and YAP (Santa Cruz, SC-101199, 1 : 250) were introduced *via* the same solution and left on for 1 hour at room temperature or overnight at 4 degrees. The plates were washed with PBS and the same was carried out for the secondary antibodies (Alexa fluor conjugated goat anti-mouse or anti-rabbit, 1 : 500) for 1 hour at room temp in PBS. Hoechst stain was added post-secondary (1 : 500) to stain DNA. Plates were imaged as above using the Opera Cell:Explorer with 20× objective lens (NA = 0.45).

### C. Image acquisition and feature extraction

Image acquisition and cell segmentation was performed using Columbus high-content image analysis software or Harmony software. Nuclei were segmented using the Hoechst channel. Cell bodies were segmented using the tubulin channel. The perinuclear region was used to measure cytoplasmic antibody intensities. The cell–cell contact area (neighbour fraction) was determined using an inbuilt Columbus algorithm ‘cell contact area with neighbors [%]’ expressed as the percent of the object border that touches a neighbor object. The border objects were removed from the analysed cells considering only cells completely imaged. Mitotic cells were filtered using a combination of Hoechst intensity mean and Hoechst intensity maximum and excluded of all the analysis of this study. Geometric features measured include: the area of all subcellular regions; the length, width, and elongation (length/width) of the cell and nucleus, cell and nuclear roundness and nucleus area/cytoplasm area.

### D. Scaling analysis

The YAP – size relationship in each DNA-cluster (either side of the median integrated Hoescht intensity) was treated as a power law such that:[*Y*] = *aA*^*b*^where; [*Y*] represents YAP concentration, ‘*A*’ cell area, and ‘*a*’ and ‘*b*’ are constants. The scaling factor, ‘*b*’ and ‘log(*a*)’ was extracted by conducting a linear fit on:log([*Y*]) = log(*a*) + *b* log(*A*)

Which results from a simple manipulation. The factor by which ‘*a*’ increases across DNA groups is trivially retrieved by:log_2_(*a*_*n*+1_) − log_2_(*a*_*n*_) = *x*, *a*_*n*+1_/*a*_*n*_ = 2^*x*^

Examining the logarithmic derivative of [*Y*], we noticed that ‘*b*’ was not constant across the entire size range captured in our populations, although was over an 8-fold size difference about the mean. To avoid this complicating our analysis, we conducted the linear fits on the data within three standard deviations of the mean. Linear fitting, clustering, and data handling were conducted in the MATLAB R2019b (MathWorks) environment.

### E. PLSR and hit detection

Regression analyses were conducted with the MATLAB (MathWorks) environment using the plsregress function from the machine learning toolbox. Partial least squares regression was selected as the method to help mitigate the influence of co-linearity in the predictor dataset. Model components were selected through 10-fold cross-validation using the elbow method on the mean square error as a function of component number. Prior to model construction, phosphopeptide abundances were adjusted to reflect ‘excess’ phosphorylation given the total expression of the peptide. To do this, a linear fit was constructed relating scaled phosphopeptide and peptide abundance across all cell lines for each gene (trivially you would expect a linear relationship). We could then calculate the deviation of the phosphopeptide abundance on a per cell line basis, by subtracting the the expected abundance given total peptide abundance from the fit. This then gives us an ‘excess’ abundance for each line. A cartoon summary of the procedure is provided in the supplement (Fig. S3, ESI†).

The influence a feature has on a model was estimated through ‘variable importance to projection’ (VIP) scores calculated as:
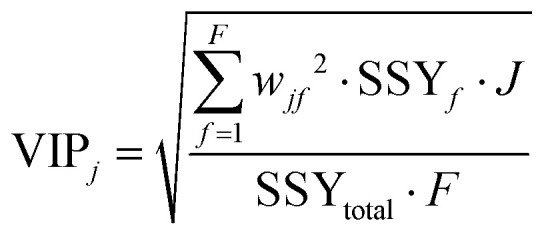
where *w*_*jf*_ is the weight value for the *j* variable and *f* component, SSY_*f*_ is the sum of squares of explained variance for the *f*th component, *J* number of *X* variables, and SSY_total_ is the total sum of squares explained of the dependent variable, and *F* is the total number of components. Features with a VIP score greater than 1 were taken as major drivers of the models.

### F. Linear classifier

For the cell cycle experiments in MCF10A cells (engineered to express endogenous mRuby-tagged PCNA) we used a manually trained linear classifier. Cell cycle classification was performed using Columbus (PerkinElmer). We used a combination of thresholding and linear classifiers based on nuclear morphology and DNA, CCNA2, and PCNA intensity and texture features. Classification was performed sequentially by manual annotation to divide and further subdivide cell cycle stages. First, nuclei and cell bodies were segmented using the DNA and YAP channels and cells touching the border were removed. Then mitotic nuclei were distinguished from interphase nuclei based primarily on DNA, PCNA and morphology features using a manually trained linear classifier (most relevant features: nucleus DNA texture bright/edge/ridge, PCNA intensity, nucleus area/roundness/width, nucleus DNA intensity). Interphase nuclei were thresholded based on mean nuclear PCNA intensity, with PCNA− nuclei classed as G0. PCNA+ nuclei were divided into CCNA2+ and CCNA2− subpopulations based on mean nuclear CCNA2 intensity and PCNA+/CCNA2− cells were classed as G1. PCNA+/CCNA2+ cells with low mean CCNA2 intensity (first quartile) were classed as early S-phase. The remaining cells were finally divided into S and G2 classes using a manually trained linear classifier. During S-phase, PCNA goes from being uniformly distributed in the nucleus to having a progressively more punctate or spotty appearance as DNA replication proceeds. The PCNA texture linear classifier was manually trained on PCNA+/CCNA2 high cells (most relevant features: PCNA texture edge/saddle/ridge/Haralick homogeneity/CV, mean nuclear CCNA2 intensity, mean perinuclear ring region CCNA2 intensity). “Spotty” nuclei classed as S-phase and “smooth” nuclei classed as G2. YAP intensity features were not included in the spotty/smooth linear classifier. Integrated DNA intensity (*i.e.* total amount of DNA) was not included in the spotty/smooth linear classifier but was used *post hoc* to verify S *versus* G2 classification.

### G. Cell preparation and proteomics analysis of the breast cell lines

Cells were plated at day 0 as stated above and collected 72 h later by trypsinization. After resuspension in growth media and centrifugation, media was removed and 1 ml of cold PBS was added. Then one million of viable cells per cell line (by duplicate) was transferred to low binding tubes and washed 2× with cold PBS to a final pellet that was flash frozen with 70% ethanol and dry ice. Cell pellets were lysed in 1% sodium deoxycholate (SDC), 100 mM triethylammonium bicarbonate (TEAB), 10% isopropanol, 50 mM NaCl buffer freshly supplemented with Halt protease and phosphatase inhibitor cocktail (100×) (Thermo, #78442), 5 mM tris-2-carboxyethyl phosphine (TCEP), 10 mM iodoacetamide (IAA) and Universal Nuclease (Pierce) followed by bath sonication for 5 min and incubation at room temperature for 45 min. Protein concentration was measured with the Quick Start Bradford protein assay. Aliquots of 60 g of total protein were digested overnight with trypsin (Pierce, ratio 1 : 20) and labelled with the TMTpro multiplex reagents (Thermo) according to manufacturers instructions. The peptide mixture was fractionated with high pH reversed-phase (RP) chromatography using the XBridge C18 column (2.1 × 150 mm, 3.5 m, Waters) on an UltiMate 3000 HPLC system over a 1% gradient in 35 min. Mobile phase A was 0.1% (v/v) ammonium hydroxide and mobile phase B was 0.1% ammonium hydroxide (v/v) in acetonitrile. Phosphopeptide enrichment was performed with the High-Select™ Fe-NTA phosphopeptide enrichment kit (Thermo) using a modified protocol in a well plate array format. A volume of 50 L resin/buffer was transferred on top of 10 L filter tips that were fitted on a 96-well plate using a suitable tip rack. The resin was washed three times with 40 L wash/binding solution and centrifugation at 500 g for 1 min. Peptides were reconstituted in 30 L wash/binding solution and were loaded onto the tip-columns with the resin. After 30 min, the flow-through (FT) from three washes with wash/binding solution were collected in a clean 96-well plate with centrifugation at 500 g for 1 min each time. Phosphopeptides were eluted twice with 40 L elution buffer in a clean 96-well plate with centrifugation at 500 g for 1 min, transferred in glass vials (Waters, P/N 186005669CV) and SpeedVac dried. Both the flow-through solutions and IMAC eluents were subjected to LC-MS analysis for bulk proteome and phosphoproteome analysis respectively. LC-MS analysis was performed on an UltiMate 3000 system coupled with the Orbitrap Fusion Lumos Mass Spectrometer (Thermo) using an Acclaim PepMap, 75 m × 50 cm C18 capillary column over a 95 min (FT) or 65 min (IMAC elution) gradient. MS spectra were collected with mass resolution of 120k and precursors were targeted for HCD fragmentation in the top speed mode with collision energy 36% and IT 54 ms (FT) or 100 ms (IMAC elution) at 30k Orbitrap resolution. Targeted precursors were dynamically excluded from further activation for 45 or 30 seconds. The Sequest HT engine in Proteome Discoverer 2.4 (Thermo) was used to search the raw mass spectra against reviewed UniProt human proteins. The precursor mass tolerance was set at 20 ppm and the fragment ion mass tolerance was 0.02 Da. TMTpro at N-terminus/K and carbamidomethyl at C were defined as static modifications. Dynamic modifications were oxidation of M and deamidation of N/Q as well as phosphorylation of S/T/Y for the phosphoproteome analysis. Peptide confidence was estimated with the Percolator node and peptide FDR was set at 0.01. Only unique peptides were used for quantification, considering protein groups for peptide uniqueness. Peptides with average reporter signal-to-noise greater than 3 were used for protein quantification.

### H. Gene set enrichment analysis

GSEA was conducted using the ‘WebGestalt’ web application on our ranked list of peptides (VIP-Score defined the rank).^94^ We used the ‘pathway’ and ‘noRedundent Biological process’ enrichment categories to identify enriched themes/pathways in the high and low scoring peptides. Parameters used: minimum IDs per category = 5, max = 10 000, permutations = 1000. Enrichments with a false discovery rate < 0.05 were taken as ‘hit’ themes and/or pathways.

### I. Drug treatments

231 and LM2 were plated in 384 wells and treated with 10 μM of binimetinib or DMSO. 24 h after cells were fixed in pre-warmed 4% formaldehyde (ThermoScientific) in PBS for 15 min at room temperature. For the palbociclib experiments in LM2, the drug was used at 0.5 μM for 24 h hours prior to formaldehyde fixation, immunofluorescence, and image analysis.

For the experiments in melanoma cells and RPEs 0.33 μM palbociclib and 0.25 μM binimetinib were added to the cell cultures and incubated for a duration of 72 hours. Following the treatment, the cells were fixed in 4% paraformaldehyde (PFA) for 15 minutes at room temperature. Primary antibody staining was performed using a dilution of 1 : 1000 for YAP and 1 : 1000 for pRB. Secondary antibody staining was conducted using a dilution of 1 : 500. All antibody stains were incubated overnight at 4 degrees celsius.

## Author contributions

I. Jones analysed the data, developed the computational models and with C. Bakal wrote the manuscript. J. Sero, M. Arias-Garcia and M. Beykou cultured the breast cells and generated the imaging datasets. J. Sero and M. Arias-Garcia carried out the image analysis. P. Pascual-Vargas conducted the RNAi experiments and performed the image analysis and M. Arias-Garcia optimised the screening and staining methodology. M. Arias-Garcia and L. Dent performed the drug experiments and their image analysis in breast and melanoma cells respectively. T. Roumeliotis, M. Beykou and M. Arias-Garcia, with J. Choudhary, generated the proteomic datasets. C. Bakal conceived and designed the research.

## Data availability

Image datasets for the cell lines used for morphological profiling are available from: DRYAD: https://dx.doi.org/10.5061/dryad.tc5g4. Image Data Repository (https://idr-demo.openmicroscopy.org/about, accession number S-BSMS6). Biostudies database (https://www.ebi.ac.uk/biostudies/studies/S-BSMS6). Proteomic/phospho-proteomic data available on the PRIDE database: Submission details: Project Name: Proteomic profiling of Breast cell lines exhibiting epithelial or mesenchymal morphology Project accession: PXD047164.

## Conflicts of interest

The authors declare no competing interests.

## Supplementary Material

MO-020-D4MO00100A-s001

MO-020-D4MO00100A-s002

MO-020-D4MO00100A-s003

MO-020-D4MO00100A-s004
