# Supplementary material for: YAP activation is robust to dilution
Source: Mol Omics. 2024 Aug 14;20(9):554–69. doi: 10.1039/d4mo00100a (PMC11403994; doi:10.1039/d4mo00100a)
Supplement: MO-020-D4MO00100A-s001 [file MO-020-D4MO00100A-s001.pdf]

Supplementary Information (SI) for Molecular Omics.
This journal is © The Royal Society of Chemistry 2024

Supplemental Information

A guide to the datasets used in this study:

- YAP concentration decreases with increasing cell size: Sero et al, mol sys bio, 2015
- YAP concentration, but not scaling, is sensitive to DNA-content and cell cycle progression: Sero et al, mol sys bio, 2015 / Sero novel to this study
- A constant nuclear concentration of YAP is maintained across cell sizes despite whole cell dilution: Sero et al, mol sys bio, 2015
- the nuclear YAP concentration is associated with altered RAS and nuclear transport signalling processes: M.Arias-Garcia, M.Beykou, novel to this study
- YAP dilution behavior is conserved across melanoma and RPE cells: L.Dent, T. Pal Chaudhuri, novel to this study
- 231/LM2 after the MEKi and LM2 after the CDK4/6 inhibitor. M.Arias-Garcia, novel to this study
- YAP RNAi experiments: P.Pascual-Vargas, Scientific data, 2017

A. YAP abundance and concentration scaling but not N/C ratio are insensitive to cell crowding. Cell size and YAP activation have previously been associated with cell crowding, cell-cell adhesion, and contact inhibition. In our cell lines, cell area negatively correlated with neighbour fraction (NF), raising the possibility that the observed YAP sub-scaling was driven by the NF (Supp.Fig.2). To investigate this, we first quantified the extent to which YAP scaled with NF, noticing a strong positive association at high NF's (0.4 -1), in accordance with the area correlation. However, when clustering the cells on NF, and conducting a scaling analysis between YAP concentration and cell area within each NF group, we observed no change in the YAP concentration per size or 'b' across clusters (Supp.Fig.3). From these data, we concluded it is the NF's influence on cell area that is driving the NF-YAP scaling relationship rather than any direct effect of the neighbour fraction. We conducted the same analysis for actin abundance, noting the same effect. Interestingly, in several lines the YAP N/C ratio exhibited a NF sensitivity (Supp.Fig.3).

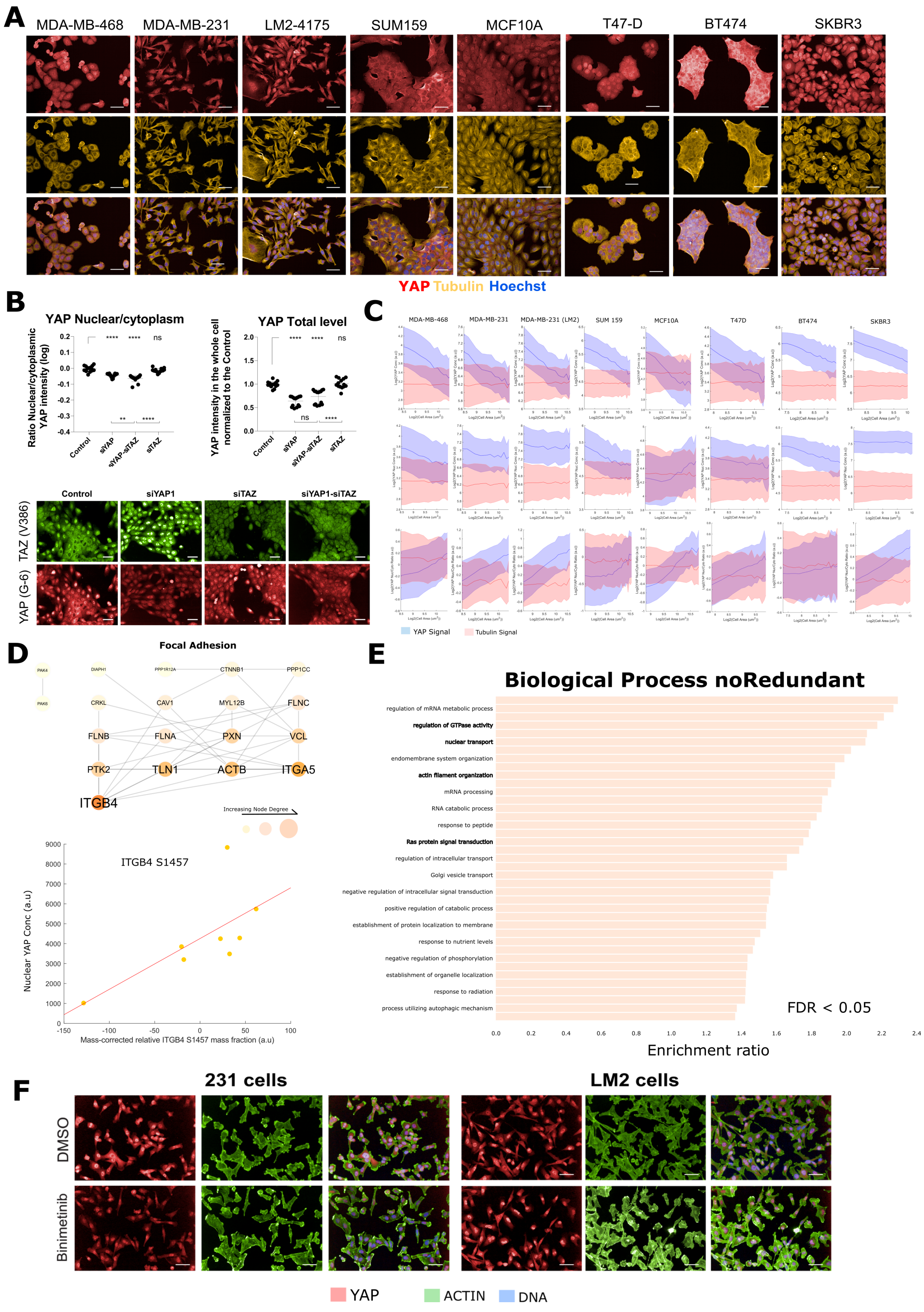

S.Figure 1: Representative images of the 8-lines across which we conducted phospho-proteomic experiments. Scale bar denotes 50um, YAP in red, tubulin in yellow, DNA stain in blue. B) Validation of the YAP (G-6) antibody used in this imaging. (left) Quantification of the YAP nuc/cyto ratio using the YAP G-6 antibody under various knockdown conditions. TAZ is included here due to the common overlap in antibody specificity. (Right) Quantification of the YAP whole cell intensity using the YAP G-6 antibody under various knockdown conditions. TAZ is included here due to the common overlap in antibody specificity. (Bottom) representative images from each treatment. C) Recapitulation of whole cell YAP dilution with increasing cell size and perfectly scaling nuclear concentration against a tubulin standard in a separate experiment and cell line panel. The central line denotes the mean YAP cytoplasmic concentration (TOP), nuclear concentration (MID) or N/C ratio (BOT) in each size bin. The error bars correspond to one standard deviation in that bin. Tubulin signal is shown in red and YAP in blue. D) A network of the interacting members of the phosphopeptides predictive of the nuclear YAP concentration under the 'Focal Adhesion' KEGG pathway. Interactions were derived from the STRING database, only experimentally determined physical interactions are shown. Node size, label size and colour are proportional to the node degree. (Bottom) Example relationships between the nuclear YAPconcentration and enriched phosphopeptides from the 'Focal Adhesion' KEGG pathway. E) Themes from the biological process noRedundent dataset enriched in the list of phosphopeptides most predictive of a cells nuclear:cytoplasmic YAP ratio. All enrichments are significant to $FDR < 0.05$. F) Representative images demonstarting the effects of binimetinib treatment on YAP abundance and translocation. Scale bar denotes 50um, YAP in red, actin in green, DNA stain in blue.

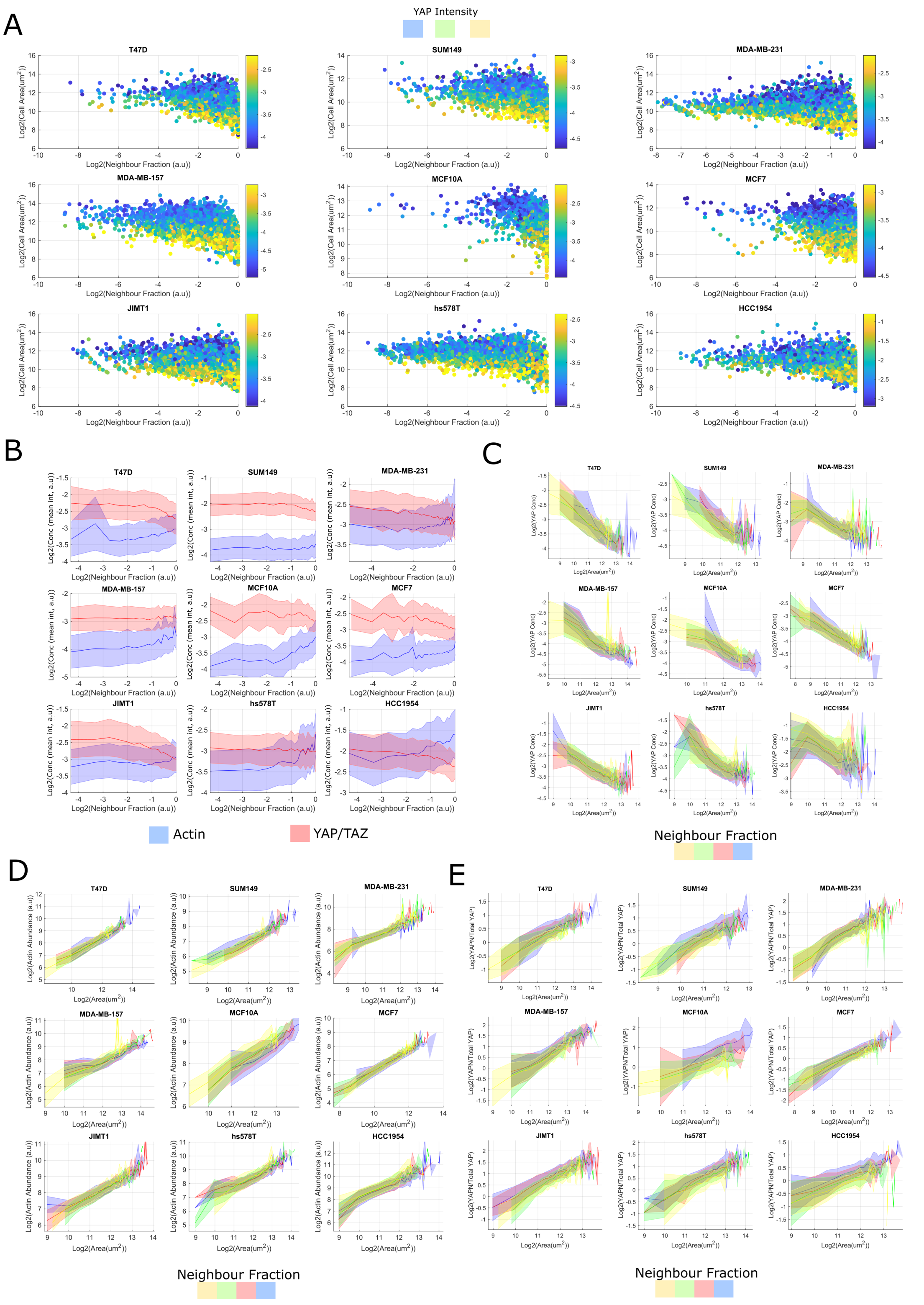

S.Figure 2: The effect of neighbour fraction on YAPscaling A) Log-log plots relating single cell neighbour fraction and cell area. Colour is proportional to the mean YAP intensity (concentration). Area and NF negatively correlate at high (close to 1) neighbour fractions. B) Log-log plots relating YAP/TAZ (blue) and Actin (red) concentration to single-cell neighbour fraction. Assuming a power-law relationship between protein concentration and cell size, the gradient represents the power to which concentration scales with NF and the y-intercept determines the initial concentration at NF = 0. The shaded area denotes one standard deviation of the cell size distribution about that size bin. C) Log-log plots relating YAP/TAZ concentration and single cell area across lines and each NF bin (lower 25%, 25 -50... etc). Blue represents the lowest NF, then red, green and yellow, the most. The shaded area denotes one standard deviation of the cell size distribution about that size bin. D/E) As in ‘C’ but relating to YAP abundance (D) or N/C ratio (E).

A

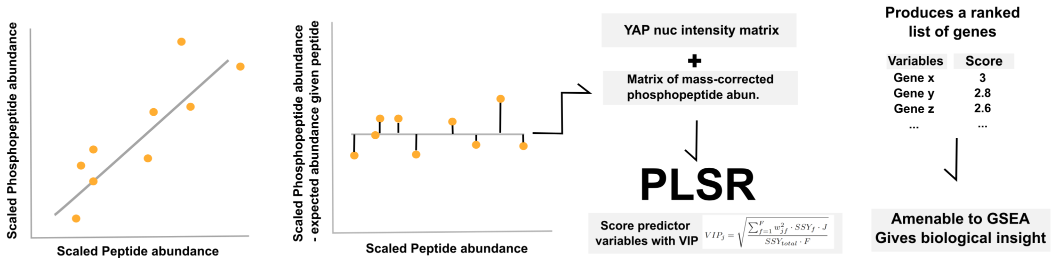

S.Figure 3: Cartoon schematic depicting the basic workflow of the proteomic analysis. A linear model built predicting the scaled abundance of a phosphopeptide in each line from the corresponding peptide abundance. The fitted values are then subtracted from the phosphopeptide abundances to get an 'excess phosphorylation'. These are used to construct a PLSR model to predict YAP nuclear intensity across lines. We use the VIP score to assess how much the abundance of any one phosphopeptide contributes to the model. This provides a ranked list which we may use in down stream ontological analyses.

Table 1. Actin Concentration Scaling: Scaling parameter values for each cell line across DNA bins within each 'K', the cluster number used in kmeans clustering on the integrated Hoechst intensity. Log(a) is proportional to the initial Actin concentration and b is the power to which Actin concentration scales with cell area.

DNA bin	$K = 1$		$K = 2$		$K = 3$		$K = 4$	
	log(a)	b	log(a)	b	log(a)	b	log(a)	b
T47D 1	-3.3179	0.0687	-3.2756	0.0641	-3.4727	0.0805	-3.4144	0.0737
2	0	0	-2.9920	0.0443	-2.7778	0.0216	-2.7935	0.0242
3	0	0	0	0	-3.1767	0.0597	-2.8385	0.0252
4	0	0	0	0	0	0	-3.3132	0.0714
SUM149 1	-0.9714	-0.1097	-0.9001	-0.1191	-0.9215	-0.1148	-0.8821	-0.1215
2	0	0	-0.2076	-0.1721	-0.5756	-0.1302	-0.2379	-0.1701
3	0	0	0	0	1.0844	0.6098	-1.0573	-0.0913
4	0	0	0	0	0	0	1.0411	0.6147
MDM MB231 1	-2.2489	-0.0540	-2.2831	-0.0530	-2.2669	-0.0549	-2.2688	-0.0548
2	0	0	-1.5399	-0.1158	-1.8311	-0.0886	-1.7991	-0.0917
3	0	0	0	0	-1.7875	-0.0975	-1.6739	-0.1078
4	0	0	0	0	0	0	-3.8237	0.0631
MDA MB157 1	-2.0089	-0.0793	-1.8068	-0.0989	-1.8722	-0.0991	-1.8623	-0.1003
2	0	0	-0.1318	-0.2102	-0.4986	-0.1980	-0.3186	-0.2154
3	0	0	0	0	-0.1666	-0.2061	-0.6992	-0.1698
4	0	0	0	0	0	0	0.1456	-0.2286
MCF10A 1	-2.9218	0.0418	-2.8995	0.0397	-3.0518	0.0543	-3.0728	0.0558
2	0	0	-3.3217	0.0782	-2.8176	0.0319	-2.7912	0.0300
3	0	0	0	0	-3.2700	0.0738	-3.2384	0.0717
4	0	0	0	0	0	0	-7.8482	0.4530
MCF7 1	-3.3127	0.0383	-3.3151	0.0390	-3.3169	0.0392	-3.3723	0.0450
2	0	0	-3.4741	0.0520	-3.4073	0.0461	-3.2827	0.0339
3	0	0	0	0	-4.5712	0.1516	-3.3871	0.0446
4	0	0	0	0	0	0	-4.9569	0.1848
JIMT1 1	-2.0891	-0.0646	-2.0323	-0.0687	-2.0284	-0.0690	-1.9776	-0.0690
2	0	0	-3.5197	0.0557	-3.4107	0.0467	-2.9770	0.0122
3	0	0	0	0	-5.5725	0.2204	-3.5962	0.0617
4	0	0	0	0	0	0	-5.3968	0.2053
hs578T 1	-1.7803	-0.1039	-1.7005	-0.1136	-1.7038	-0.1134	-1.6932	-0.1149
2	0	0	-0.6294	-0.1903	-0.5262	-0.1996	-0.5156	-0.2057
3	0	0	0	0	-1.4700	-0.1092	-0.6504	-0.1871
4	0	0	0	0	0	0	-1.1401	-0.1339
HCC1954 1	-2.0209	-0.0155	-2.2562	0.0079	-2.2716	0.0093	-2.2354	0.0064
2	0	0	-1.9495	-0.0249	-1.8453	-0.0326	-2.5514	0.0337
3	0	0	0	0	-4.0674	0.1412	-2.0490	-0.0183
4	0	0	0	0	0	0	-4.1502	0.1473

Table 2. YAP Concentration Scaling: Scaling parameter values for each cell line across DNA bins within each 'K', the cluster number used in kmeans clustering on the integrated Hoechst intensity. Log(a) is proportional to the initial YAP concentration and b is the power to which YAP concentration scales with cell area.

DNA bin	$K = 1$		$K = 2$		$K = 3$		$K = 4$	
	log(a)	b	log(a)	b	log(a)	b	log(a)	b
T47D 1	0.5262	-0.3407	0.8357	-0.3736	0.7975	-0.3786	0.8452	-0.3840
2	0	0	2.9510	-0.5278	1.8227	-0.4566	1.8826	-0.4631
3	0	0	0	0	3.0933	-0.5390	3.1395	-0.5505
4	0	0	0	0	0	0	3.1135	-0.5360
SUM149 1	-0.1526	-0.3459	0.1584	-0.3817	0.1982	-0.3868	0.1983	-0.3868
2	0	0	0.7172	-0.4077	1.0614	-0.4432	1.1188	-0.4490
3	0	0	0	0	1.2846	-0.4076	1.7730	-0.4523
4	0	0	0	0	0	0	45.6573	-3.6959
MDM MB231 1	1.3997	-0.4333	1.9765	-0.4994	2.0367	-0.5074	2.0521	-0.5093
2	0	0	2.3003	-0.4980	3.0583	-0.5729	3.1612	-0.5831
3	0	0	0	0	2.1716	-0.4440	2.8563	-0.5097
4	0	0	0	0	0	0	4.9784	-0.5895
MDA MB157 1	0.9423	-0.4199	1.3804	-0.4621	1.2999	-0.4673	1.3195	-0.4697
2	0	0	3.8252	-0.6085	3.7629	-0.6407	4.0220	-0.6665
3	0	0	0	0	4.3659	-0.6457	4.0844	-0.6426
4	0	0	0	0	0	0	4.5864	-0.6578
MCF10A 1	1.6437	-0.4253	1.5918	-0.4234	1.3702	-0.4065	1.3040	-0.4004
2	0	0	2.9101	-0.5091	1.9159	-0.4493	1.9286	-0.4507
3	0	0	0	0	2.8531	-0.5036	2.9763	-0.5163
4	0	0	0	0	0	0	-2.1396	-0.0434
MCF7 1	-0.3921	-0.3218	-0.1148	-0.3548	-0.1139	-0.3555	-0.1103	-0.3570
2	0	0	0.7019	-0.4104	0.8025	-0.4229	0.8121	-0.4294
3	0	0	0	0	1.4729	-0.4475	1.0325	-0.4406
4	0	0	0	0	0	0	1.5877	-0.4555
JIMT1 1	1.2772	-0.4002	1.9080	-0.4663	2.3463	-0.5182	2.3612	-0.5201
2	0	0	3.4679	-0.5685	2.9729	-0.5514	2.9728	-0.5520
3	0	0	0	0	3.5474	-0.5726	3.8726	-0.6028
4	0	0	0	0	0	0	2.6220	-0.4405
hs578T 1	1.0447	-0.3858	1.4946	-0.4327	1.5253	-0.4357	1.5123	-0.4376
2	0	0	2.9914	-0.5216	3.2113	-0.5420	3.4200	-0.5822
3	0	0	0	0	4.4841	-0.5914	3.1357	-0.5330
4	0	0	0	0	0	0	4.4841	-0.5914
HCC1954 1	1.6923	-0.3397	2.2352	-0.3970	2.3170	-0.4061	2.1364	-0.3907
2	0	0	2.7372	-0.4109	3.5686	-0.4888	4.3223	-0.5697
3	0	0	0	0	2.9610	-0.3894	3.2340	-0.4526
4	0	0	0	0	0	0	2.8783	-0.3839

Table 3. YAP Abundance Scaling: Scaling parameter values for each cell line across DNA bins within each 'K', the cluster number used in kmeans clustering on the integrated Hoechst intensity. Log(a) is proportional to the initial YAP abundance and b is the power to which YAP abundance scales with cell area.

DNA bin	$K = 1$		$K = 2$		$K = 3$		$K = 4$	
	log(a)	b	log(a)	b	log(a)	b	log(a)	b
T47D 1	0.4277	0.6686	0.7347	0.6361	0.6903	0.6323	0.7322	0.6276
2	0	0	2.1366	0.5428	1.5646	0.5671	1.6178	0.5612
3	0	0	0	0	2.1900	0.5392	2.4472	0.5098
4	0	0	0	0	0	0	1.9505	0.5645
SUM149 1	-0.4643	0.6847	0.0267	0.6314	0.0821	0.6248	0.0825	0.6248
2	0	0	0.0597	0.6538	0.8467	0.5770	0.8475	0.5767
3	0	0	0	0	1.0844	0.6098	1.4860	0.5738
4	0	0	0	0	0	0	2.6006	-0.6672
MDM MB231 1	1.0130	0.6044	1.9090	0.5073	1.9331	0.5046	1.9596	0.4975
2	0	0	1.6388	0.5645	1.8648	0.5429	3.1963	0.4086
3	0	0	0	0	3.3455	0.5263	2.2313	0.5101
4	0	0	0	0	0	0	1.9114	0.5850
MDA MB157 1	0.6812	0.6033	1.1719	0.5567	1.2103	0.5410	1.2320	0.5387
2	0	0	3.4952	0.4183	3.4677	0.3848	3.5225	0.3788
3	0	0	0	0	3.9458	0.3882	4.1585	0.3580
4	0	0	0	0	0	0	4.2793	0.3727
MCF10A 1	1.6390	0.5751	1.6480	0.5714	1.6498	0.5713	1.6498	0.5713
2	0	0	2.8037	0.5003	2.7770	0.5003	2.7878	0.4994
3	0	0	0	0	2.1516	0.5918	4.5335	0.3911
4	0	0	0	0	0	0	-6.0728	1.2847
MCF7 1	-0.3816	0.6771	-0.1306	0.6468	-0.1328	0.6465	-0.1416	0.6462
2	0	0	0.6360	0.5959	0.7930	0.5780	0.7511	0.5766
3	0	0	0	0	0.9703	0.5988	1.0212	0.5604
4	0	0	0	0	0	0	1.0781	0.5912
JIMT1 1	1.1222	0.6145	1.8581	0.5386	2.2526	0.4911	2.2744	0.4886
2	0	0	2.8276	0.4879	2.8270	0.4622	2.8235	0.4619
3	0	0	0	0	2.7701	0.4955	3.3382	0.4442
4	0	0	0	0	0	0	2.2030	0.5939
hs578T 1	0.7421	0.6410	1.3691	0.5786	1.4070	0.5749	1.4215	0.5706
2	0	0	2.4033	0.5281	2.7356	0.4983	2.7658	0.4733
3	0	0	0	0	4.2205	0.4298	2.6690	0.5064
4	0	0	0	0	0	0	4.2205	0.4298
HCC1954 1	1.4279	0.6852	2.2160	0.6048	2.2937	0.5961	2.1937	0.6037
2	0	0	2.2640	0.6311	3.0687	0.5562	3.8376	0.4750
3	0	0	0	0	2.0141	0.6893	2.8693	0.5797
4	0	0	0	0	0	0	1.9641	0.6916

Table 4. Actin Abundance Scaling: Scaling parameter values for each cell line across DNA bins within each 'K', the cluster number used in kmeans clustering on the integrated Hoechst intensity. Log(a) is proportional to the initial Actin abundance and b is the power to which Actin abundance scales with cell area.

DNA bins	$K=1$		$K=2$		$K=3$		$K=4$	
	log(a)	b	log(a)	b	log(a)	b	log(a)	b
T47D 1	-3.2001	1.0575	-3.1339	1.0505	-3.3154	1.0644	-3.2744	1.0600
2	0	0	-2.8217	1.0296	-2.6167	1.0068	-2.6193	1.0069
3	0	0	0	0	-2.9513	1.0401	-3.0228	1.0458
4	0	0	0	0	0	0	-2.7112	1.0198
SUM149 1	-1.0025	0.8933	-0.9226	0.8853	-0.8855	0.8794	-0.8963	0.8799
2	0	0	-0.5366	0.8665	-0.4454	0.8500	-0.2041	0.8267
3	0	0	0	0	1.0844	0.6098	-1.0362	0.9068
4	0	0	0	0	0	0	2.6006	-0.6672
MDM MB231 1	-2.1327	0.9346	-2.1865	0.9374	-2.1787	0.9363	-2.1893	0.9373
2	0	0	-1.5026	0.8807	-1.6141	0.8906	-1.6281	0.8920
3	0	0	0	0	-1.8453	0.9076	-1.6817	0.8933
4	0	0	0	0	0	0	-4.4738	1.0964
MDA MB157 1	-2.0519	0.9245	-1.8712	0.9069	-1.9028	0.9037	-1.8963	0.9030
2	0	0	-0.2542	0.7998	-0.6160	0.8121	-0.6463	0.8144
3	0	0	0	0	-0.2739	0.8025	0.0133	0.7799
4	0	0	0	0	0	0	-2.8673	1.0039
MCF10A 1	-2.8750	1.0375	-2.8209	1.0325	-2.8200	1.0324	-2.9063	1.0402
2	0	0	-3.2331	1.0704	-3.2336	1.0709	-2.7583	1.0270
3	0	0	0	0	-3.7696	1.1020	-3.2170	1.0698
4	0	0	0	0	0	0	-3.8844	1.1070
MCF7 1	-3.2678	1.0338	-3.2629	1.0337	-3.2604	1.0334	-3.2881	1.0363
2	0	0	-3.4257	1.0474	-3.3604	1.0416	-3.2727	1.0329
3	0	0	0	0	-4.0509	1.1038	-3.3499	1.0411
4	0	0	0	0	0	0	-4.2011	1.1156
JIMT1 1	-2.1892	0.9449	-2.0989	0.9377	-2.0804	0.9360	-2.0125	0.9346
2	0	0	-3.5629	1.0595	-3.4767	1.0526	-3.0369	1.0178
3	0	0	0	0	-4.8783	1.1628	-3.6001	1.0622
4	0	0	0	0	0	0	-5.0995	1.1809
hs578T 1	-1.8602	0.9031	-1.6944	0.8859	-1.6927	0.8856	-1.6958	0.8858
2	0	0	-1.0074	0.8417	-0.8065	0.8242	-0.7820	0.8221
3	0	0	0	0	-1.7049	0.9097	-1.6399	0.9033
4	0	0	0	0	0	0	-7.0092	1.3349
HCC1954 1	-1.8894	0.9720	-1.9892	0.9821	-2.0062	0.9836	-1.9986	0.9832
2	0	0	-1.9533	0.9755	-1.8147	0.9646	-1.9456	0.9784
3	0	0	0	0	-3.5068	1.0947	-2.2007	0.9946
4	0	0	0	0	0	0	-3.5954	1.1008

Table 5. YAP Ratio Scaling: Scaling parameter values for each cell line across DNA bins within each 'K', the cluster number used in kmeans clustering on the integrated Hoechst intensity. Log(a) is proportional to the initial YAP ratio and b is the power to which YAP ratio scales with cell area.

DNA bins	$K = 1$		$K = 2$		$K = 3$		$K = 4$	
	log(a)	b	log(a)	b	log(a)	b	log(a)	b
T47D 1	-3.7184	0.3538	-3.7698	0.3593	-3.9382	0.3771	-3.9423	0.3776
2	0	0	-4.0243	0.3765	-3.7953	0.3609	-3.8076	0.3623
3	0	0	0	0	-4.0252	0.3760	-3.8289	0.3634
4	0	0	0	0	0	0	-3.9161	0.3666
SUM149 1	-4.5111	0.4383	-4.7306	0.4606	-4.7200	0.4595	-4.7100	0.4588
2	0	0	-4.2107	0.4077	-4.2781	0.4142	-4.4984	0.4349
3	0	0	0	0	1.0844	0.6098	-4.4554	0.4250
4	0	0	0	0	0	0	2.6006	-0.6672
MDM MB231 1	-4.4381	0.4709	-4.9459	0.5227	-4.9796	0.5262	-5.0023	0.5285
2	0	0	-4.0647	0.4322	-4.4357	0.4687	-4.7030	0.4967
3	0	0	0	0	-5.0151	0.4960	-4.2615	0.4497
4	0	0	0	0	0	0	-5.1602	0.5044
MDA MB157 1	-5.3866	0.5074	-5.4141	0.5103	-5.3701	0.5064	-5.3665	0.5061
2	0	0	-6.1785	0.5649	-5.5694	0.5231	-5.5672	0.5231
3	0	0	0	0	-6.0631	0.5545	-6.3036	0.5738
4	0	0	0	0	0	0	-3.6657	0.3590
MCF10A 1	-4.9326	0.4358	-4.8611	0.4297	-4.5969	0.4077	-4.5415	0.4026
2	0	0	-5.6429	0.4947	-5.1453	0.4533	-5.1558	0.4542
3	0	0	0	0	-5.6250	0.4925	-5.6539	0.4970
4	0	0	0	0	0	0	-5.2204	0.4295
MCF7 1	-4.1951	0.3976	-4.2538	0.4037	-4.2500	0.4032	-4.2170	0.3996
2	0	0	-4.0951	0.3876	-4.1438	0.3934	-4.2358	0.4037
3	0	0	0	0	-4.4485	0.4072	-4.1816	0.3956
4	0	0	0	0	0	0	-4.4507	0.4063
JIMT1 1	-5.2234	0.5266	-5.6523	0.5702	-5.8733	0.5963	-5.8993	0.5996
2	0	0	-5.7522	0.5621	-6.1314	0.6084	-6.1220	0.6084
3	0	0	0	0	-5.8167	0.5652	-6.3189	0.6151
4	0	0	0	0	0	0	-5.4792	0.5300
hs578T 1	-4.5086	0.4295	-4.7467	0.4523	-4.7635	0.4538	-4.7266	0.4506
2	0	0	-4.6766	0.4369	-4.8156	0.4496	-4.9701	0.4688
3	0	0	0	0	-5.4278	0.4730	-5.0081	0.4626
4	0	0	0	0	0	0	-5.4298	0.4732
HCC1954 1	-3.8277	0.3523	-4.0674	0.3762	-4.0849	0.3780	-4.0849	0.3780
2	0	0	-3.8424	0.3492	-4.0080	0.3656	-3.9826	0.3639
3	0	0	0	0	-5.0037	0.4273	-4.9538	0.4272
4	0	0	0	0	0	0	-7.3042	0.5841

Table 6. YAP Nuclear Scaling: Scaling parameter values for each cell line across DNA bins within each 'K', the cluster number used in kmeans clustering on the integrated Hoechst intensity. Log(a) is proportional to the initial YAP nuclear concentration and b is the power to which YAP nuclear concentration scales with cell area.

DNA bins	$K=1$		$K=2$		$K=3$		$K=4$	
	log(a)	b	log(a)	b	log(a)	b	log(a)	b
T47D 1	-3.2907	0.0224	-3.0351	-0.0046	-3.2447	0.0091	-3.2009	0.0043
2	0	0	-1.8877	-0.0807	-2.2368	-0.0715	-2.1733	-0.0782
3	0	0	0	0	-1.8368	-0.0847	-1.8376	-0.0895
4	0	0	0	0	0	0	-1.8577	-0.0792
SUM149 1	-4.9754	0.1230	-4.7030	0.0919	-4.6279	0.0836	-4.6291	0.0837
2	0	0	-4.1466	0.0612	-3.6632	0.0130	-3.6473	0.0112
3	0	0	0	0	-3.1299	0.0121	-2.9694	-0.0013
4	0	0	0	0	0	0	2.6006	-0.6672
MDM MB231 1	-3.4251	0.0754	-3.0369	0.0299	-3.0159	0.0260	-3.0021	0.0245
2	0	0	-2.4259	-0.0033	-1.6974	-0.0735	-1.6858	-0.0747
3	0	0	0	0	-3.2183	0.0843	-3.1292	0.0744
4	0	0	0	0	0	0	-3.2497	0.1093
MDA MB157 1	-4.7055	0.1107	-4.2422	0.0670	-4.1574	0.0472	-4.1500	0.0465
2	0	0	-2.6833	-0.0168	-2.1044	-0.0919	-2.1003	-0.0923
3	0	0	0	0	-2.1173	-0.0573	-2.1900	-0.0532
4	0	0	0	0	0	0	0.5188	-0.2218
MCF10A 1	-3.2935	0.0109	-3.2132	0.0011	-3.2116	0.0010	-3.1906	-0.0010
2	0	0	-2.8392	-0.0050	-2.8533	-0.0039	-3.4012	0.0450
3	0	0	0	0	-3.4502	0.0437	-2.4171	-0.0418
4	0	0	0	0	0	0	-7.8275	0.4099
MCF7 1	-4.5767	0.0747	-4.3844	0.0504	-4.3828	0.0497	-4.3587	0.0458
2	0	0	-3.4590	-0.0165	-3.3509	-0.0287	-3.4847	-0.0196
3	0	0	0	0	-3.4782	0.0061	-3.1605	-0.0439
4	0	0	0	0	0	0	-3.3726	-0.0025
JIMT1 1	-4.1012	0.1410	-3.7943	0.1088	-3.6254	0.0880	-3.6121	0.0864
2	0	0	-2.9245	0.0500	-3.3025	0.0703	-3.3069	0.0704
3	0	0	0	0	-3.0465	0.0607	-2.7758	0.0368
4	0	0	0	0	0	0	-3.3505	0.1035
hs578T 1	-3.7665	0.0705	-3.3777	0.0309	-3.3565	0.0287	-3.3388	0.0245
2	0	0	-2.2733	-0.0349	-2.0800	-0.0521	-2.1138	-0.0646
3	0	0	0	0	-1.2073	-0.0972	-2.2373	-0.0379
4	0	0	0	0	0	0	-1.2073	-0.0972
HCC1954 1	-2.3998	0.0375	-1.8529	-0.0189	-1.7912	-0.0259	-1.9462	-0.0126
2	0	0	-1.5727	-0.0201	-0.9309	-0.0790	-0.2789	-0.1468
3	0	0	0	0	-2.9907	0.1166	-1.1521	-0.0563
4	0	0	0	0	0	0	-3.3299	0.1406

Table 7. Population Characteristics: Cell area distribution statistics. 'G1' and 'G2' are defined via being above or below the median integrated Hoechst intensity

Population	G1 mean (um^2)	G1 CV (%)	G2 mean (um^2)	G2 CV (%)
MCF7	1200	0.6	1710	0.52
MDMMB231	1360	0.49	1820	0.48
T47D	1420	0.61	2760	0.56
JIMT1	1470	0.57	2390	0.49
SUM149	1480	0.57	1850	0.53
HCC1954	1840	0.55	3060	0.45
MCF10A	2660	0.63	3280	0.6
MDAMB157	3150	0.61	4760	0.56
hs578T	3280	0.47	4920	0.44
Average	N/A	0.57 ± 0.05	N/A	0.51 ± 0.05

Reagent or Resource	Source	Identifier
**Antibodies**		
YAP Antibody (G-6) -Mouse	Santa Cruz	Cat# sc-376830
YAP [67.3] -Mouse	Santa Cruz	Cat# sc-101199
TAZ (V386) Antibody -Rabbit	Cell Signalling	Cat# 4883
Tubulin Alpha Antibody — YL1/2 -Rat	Bio-Rad	Cat# MCA77G
Phospho-Rb (Ser807/811) (D20B12) XP® -Rabbit	Cell Signalling	Cat# 8516S
Recombinant Anti-Cyclin A2 antibody [EPR17351] -Rabbit	Abcam	Cat# ab181591
Goat anti-Mouse IgG (H+L) Highly Cross-Adsorbed Secondary Antibody, Alexa Fluor 488	Invitrogen	Cat# A11029
Goat anti-Rabbit IgG (H+L) Highly Cross-Adsorbed Secondary Antibody, Alexa Fluor 488	Invitrogen	Cat# A11034
Goat anti-Mouse IgG (H+L) Cross-Adsorbed Secondary Antibody, Alexa Fluor 568	Invitrogen	Cat# A11004
Goat anti-Rat IgG (H+L) Cross-Adsorbed Secondary Antibody, Alexa Fluor 568	Invitrogen	Cat# A11077
Goat anti-Mouse IgG (H+L) Cross-Adsorbed Secondary Antibody, Alexa Fluor™ 647	Invitrogen	Cat# A21235
F(ab')2-Goat anti-Rabbit IgG (H+L) Cross-Adsorbed Secondary Antibody, Alexa Fluor 647	Invitrogen	Cat# A21246
Goat anti-Rat IgG (H+L) Cross-Adsorbed Secondary Antibody, Alexa Fluor 647	Invitrogen	Cat# A21247
**Bacterial and Virus Strains**		
**Biological Samples**		
**Chemicals, Peptides, and Recombinant Proteins**		
RPMI 1640 Medium, no phenol red	GIBCO	Cat# 11835
DMEM/F-12, GlutaMAX™ Supplement	GIBCO	Cat# 31331
Horse Serum	GIBCO	Cat# 16050
Insulin	Sigma	Cat# I-1882
Cholera toxin	Sigma	Cat# C-8052
Hydrocortisone	Sigma	Cat# H-0888
Human EGF	Sigma	Cat# E-9644
Penicillin-Streptomycin	GIBCO	Cat# 15070
Human EGF	Sigma	Cat# E-9644
RPMI 1640 Medium, no phenol red	GIBCO	Cat# 11835
DMEM, high glucose, pyruvate	GIBCO	Cat# 41966
Fetal Bovine Serum (heat-inactivated)	GIBCO	Cat# 16140
Trypsin-EDTA (0.25%), phenol red	GIBCO	Cat# 25200
Opti-MEM I Reduced Serum Medium	GIBCO	Cat# 31985
Lipofectamine RNAiMAX	Invitrogen	Cat# 13778
Pierce 16% Formaldehyde (w/v), Methanol-free	Thermo Scientific	Cat# 28908
Alexa Fluor 647 Phalloidin	Invitrogen	Cat# A22287
Alexa Fluor 488 Phalloidin	Invitrogen	Cat# A-12379
Hoechst 33258	Invitrogen	Cat# H3569
Palbociclib, PD0332991	Sigma	Cat# PZ0199
Binimetinib	Sigma	Cat#
**Critical Commercial Assays**		
**Deposited Data**		
Proteome?		
**Experimental Models: Cell Lines**		
T47-D	N. Turner (ICR)	N/A
BT474	N. Turner (ICR)	N/A
MDA-MB-468	G. Poulogiannis (ICR)	N/A
SKBR3	O. Rossanese (ICR)	N/A
SUM159	R. Natrajan (ICR)	N/A
MDA-MB-231	J. Erler (University of Copenhagen)	N/A
MDA-MB-231-LM2-4175	J. Massagué (Sloan Kettering Institute) Minn, Andy J. et al., 2005	N/A
MCF10A mRuby-PCNA	J. Mansfeld (Technische Universität Dresden) Zerjatke, Thomas et al., 2017	N/A
hTERT RPE-1		
WMs		
a375p		
**Experimental Models: Organisms/Strains**		
**Oligonucleotides and siRNA (Sequenced-Based reagents)**		
**Recombinant DNA**		
**Software and Algorithms**		
Morpheus	Broad Institute	https://software.broadinstitute.org/morpheus/
Acapella 4.0	PerkinElmer	N/A
Columbus Image Data Storage and Analysis System	PerkinElmer	http://columbus2.icr.ac.uk/login?next=/
Harmony High-Content Imaging and Analysis Software	PerkinElmer	https://www.perkinelmer.com/product/harmony-5-1-office-hh17000012
Prism	GraphPad	https://www.graphpad.com/scientific-software/prism/
Excel	Microsoft	N/A
ImageJ	Schneider et al., 2012	https://imagej.nih.gov/ij/
Adobe Illustrator	Figures	http://www.adobe.com/de/products/illustrator.html, versionCC2015.3
Other		
PhenoPlate 384-well	PerkinElmer	Cat# 6057300

Table 8. List of reagents and resources.
