# Supplementary figures and images for: YAP activation is robust to dilution
Source: Mol Omics. 2024 Aug 14;20(9):554–69. doi: 10.1039/d4mo00100a (PMC11403994; doi:10.1039/d4mo00100a)

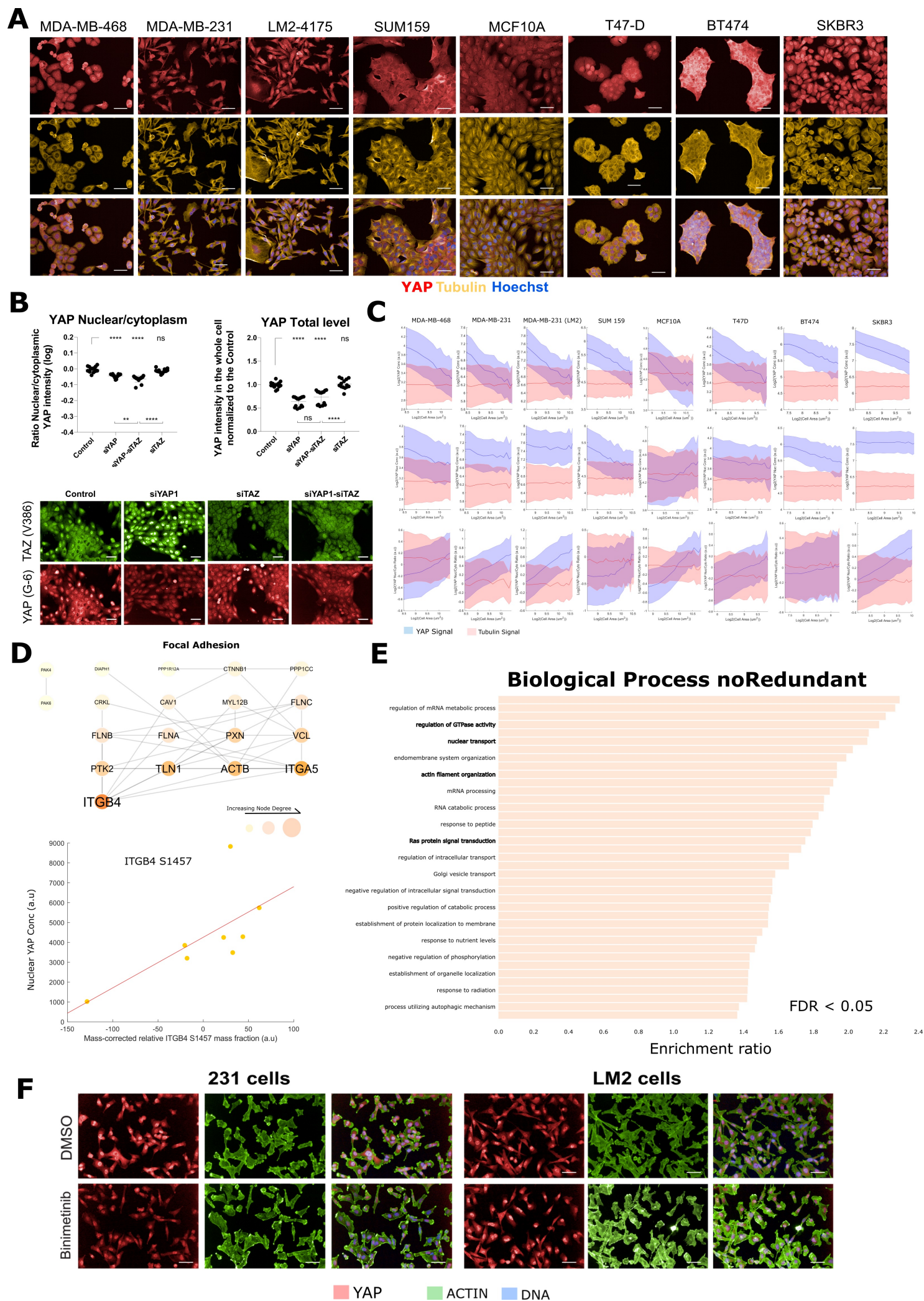

Supplement: MO-020-D4MO00100A-s002 [file MO-020-D4MO00100A-s002.pdf]

A

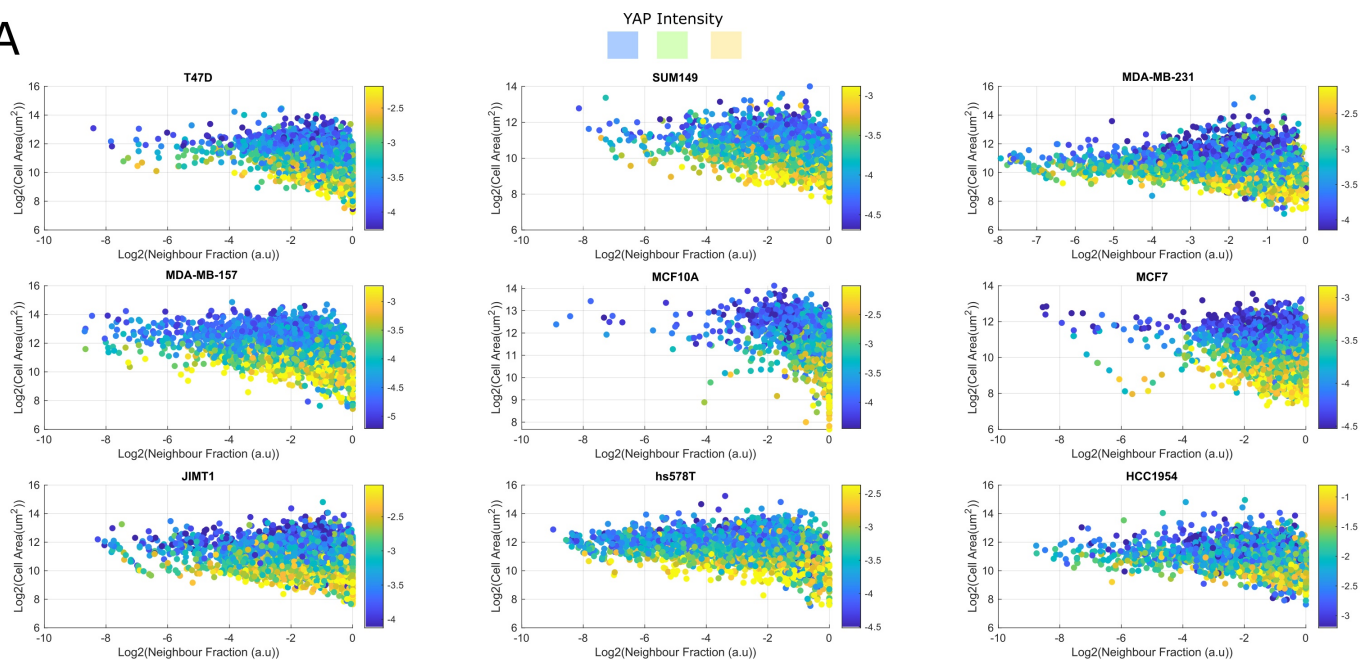

B

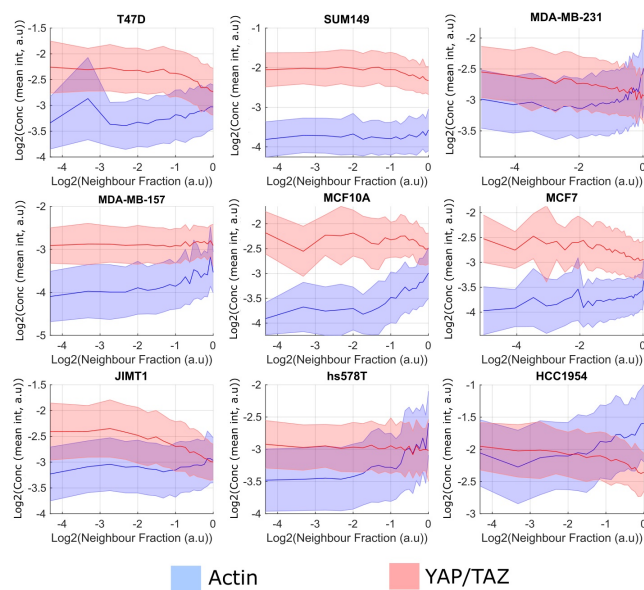

C

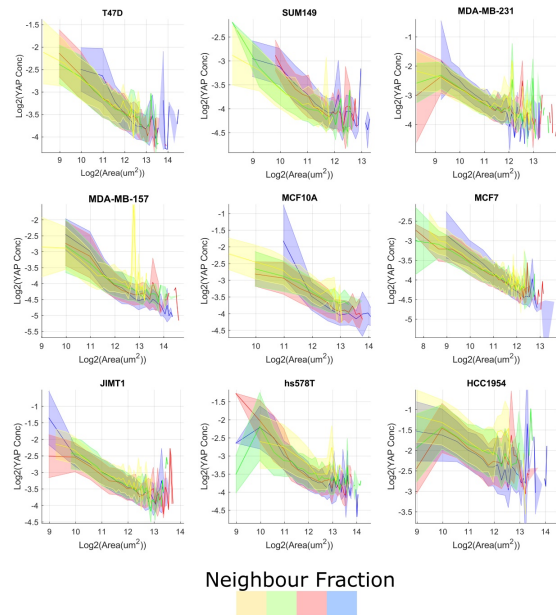

D

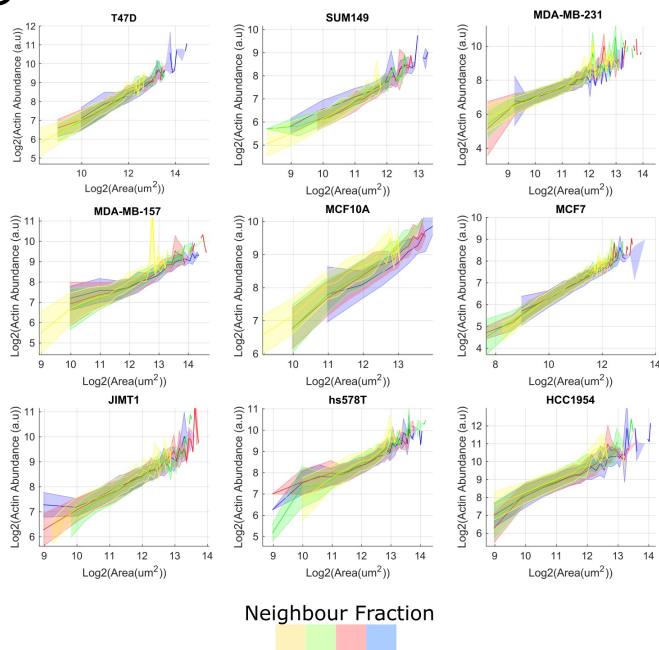

E

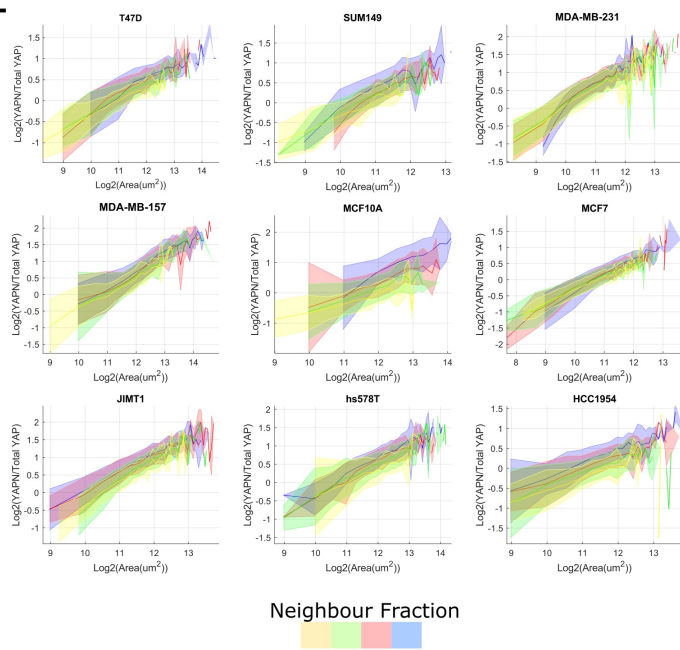

Supplement: MO-020-D4MO00100A-s003 [file MO-020-D4MO00100A-s003.pdf]
