# Supplementary material for: YAP activation is robust to dilution
Source: Mol Omics. 2024 Aug 14;20(9):554–69. doi: 10.1039/d4mo00100a (PMC11403994; doi:10.1039/d4mo00100a)

Supplementary Information (SI) for Molecular Omics.
This journal is © The Royal Society of Chemistry 2024

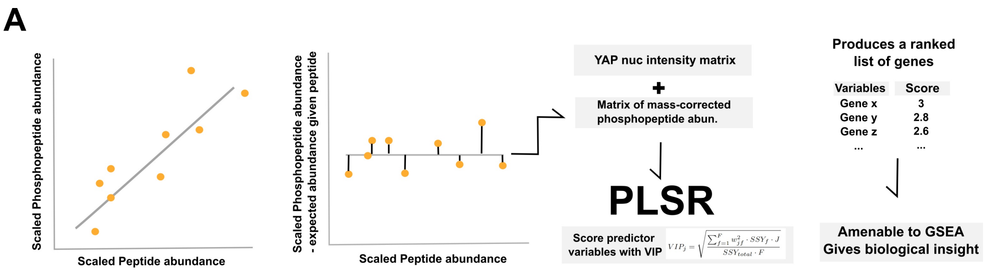

Supplement: MO-020-D4MO00100A-s004 [file MO-020-D4MO00100A-s004.pdf]
